# Supplementary material for: Transgressive and parental dominant gene expression and cytosine methylation during seed development in Brassica napus hybrids
Source: Theor Appl Genet. 2023 Apr 18;136(5):113. doi: 10.1007/s00122-023-04345-7 (PMC10113308; doi:10.1007/s00122-023-04345-7)
Supplement: Supplementary file 1 — Supplementary file1 (PDF 4957 kb) [file 122_2023_4345_MOESM1_ESM.pdf]

Supplementary Figures

Transgressive and parental dominant gene expression and cytosine methylation during seed development in *Brassica napus* hybrids

Mauricio Orantes-Bonilla[1†], Hao Wang[2†], HueyTyng Lee[1], Agnieszka A. Golicz[1], Dandan Hu[2], Wenwen Li[2], Jun Zou[2] & Rod J. Snowdon[1*]

[1] Department of Plant Breeding, IFZ Research Centre for Biosystems, Land Use and Nutrition, Justus Liebig University, Giessen, Germany

[2] National Key Laboratory of Crop Genetic Improvement, College of Plant Science & Technology, Huazhong Agricultural University, Wuhan, People's Republic of China

*** Correspondence:**

Corresponding author: Rod.Snowdon@agrar.uni-giessen.de

[†] These authors have contributed equally to this work

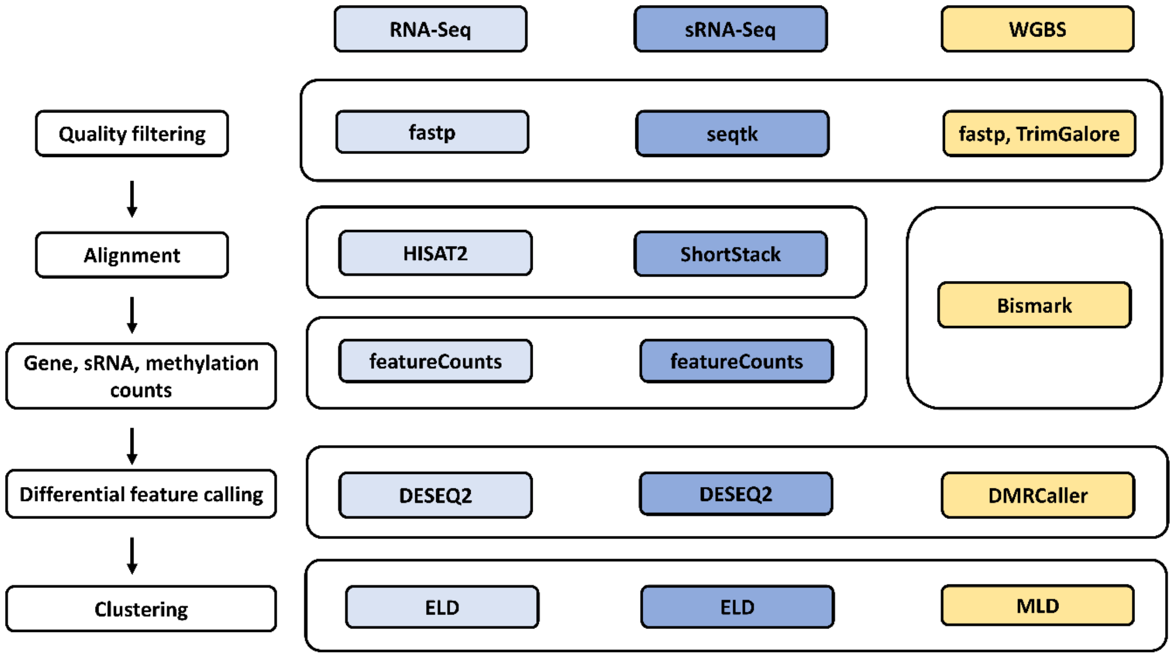

Supplementary Figure 1 Summary of main bioinformatic workflow for dominance level analyses. RNA, sRNA and WGBS workflows and tools used for each developmental stage are represented in light blue, blue and yellow respectively, while arrows indicate the workflow direction. Briefly, each biological replicate was quality filtered and then aligned to the Express 617 reference (Lee et al., 2020). The depth for each feature (genes, sRNAs and methylated cytosines) were calculated for each biological replicate and used for differentially expressed genes, siRNAs, miRNAs and differentially methylated regions calling. All features were then clustered based on how the hybrid genotype contrasted in comparison to both parents in terms of expression level dominance, and methylation level dominance (MLD).

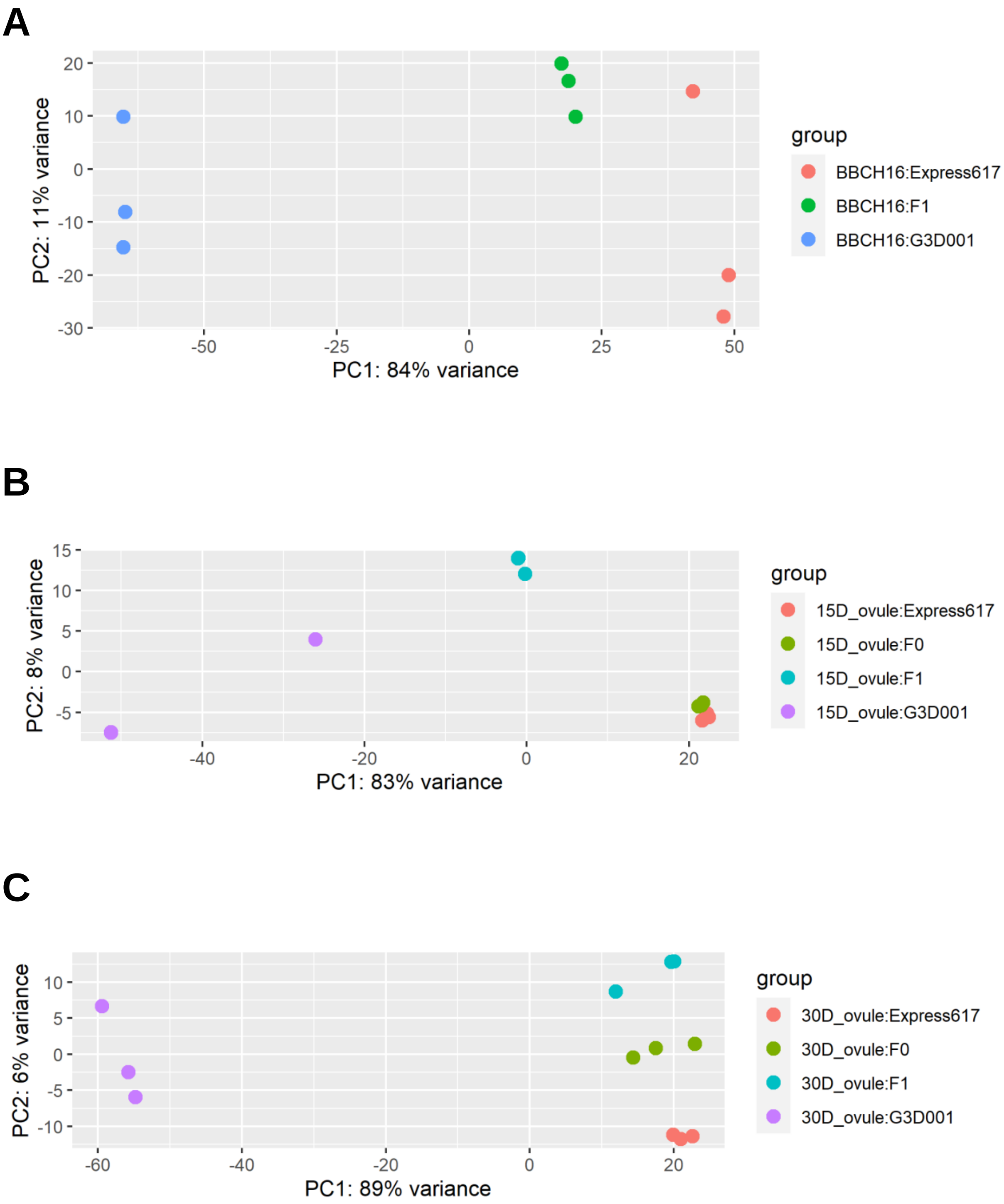

Supplementary Figure 2 Principal component analysis (PCA) of transcriptomic counts derived from RNA-Seq libraries. Each circle represents a biological replicate during **(a)** the seedling, (**b)** 15 days after pollination and **(c)** 30 days after pollination stages.

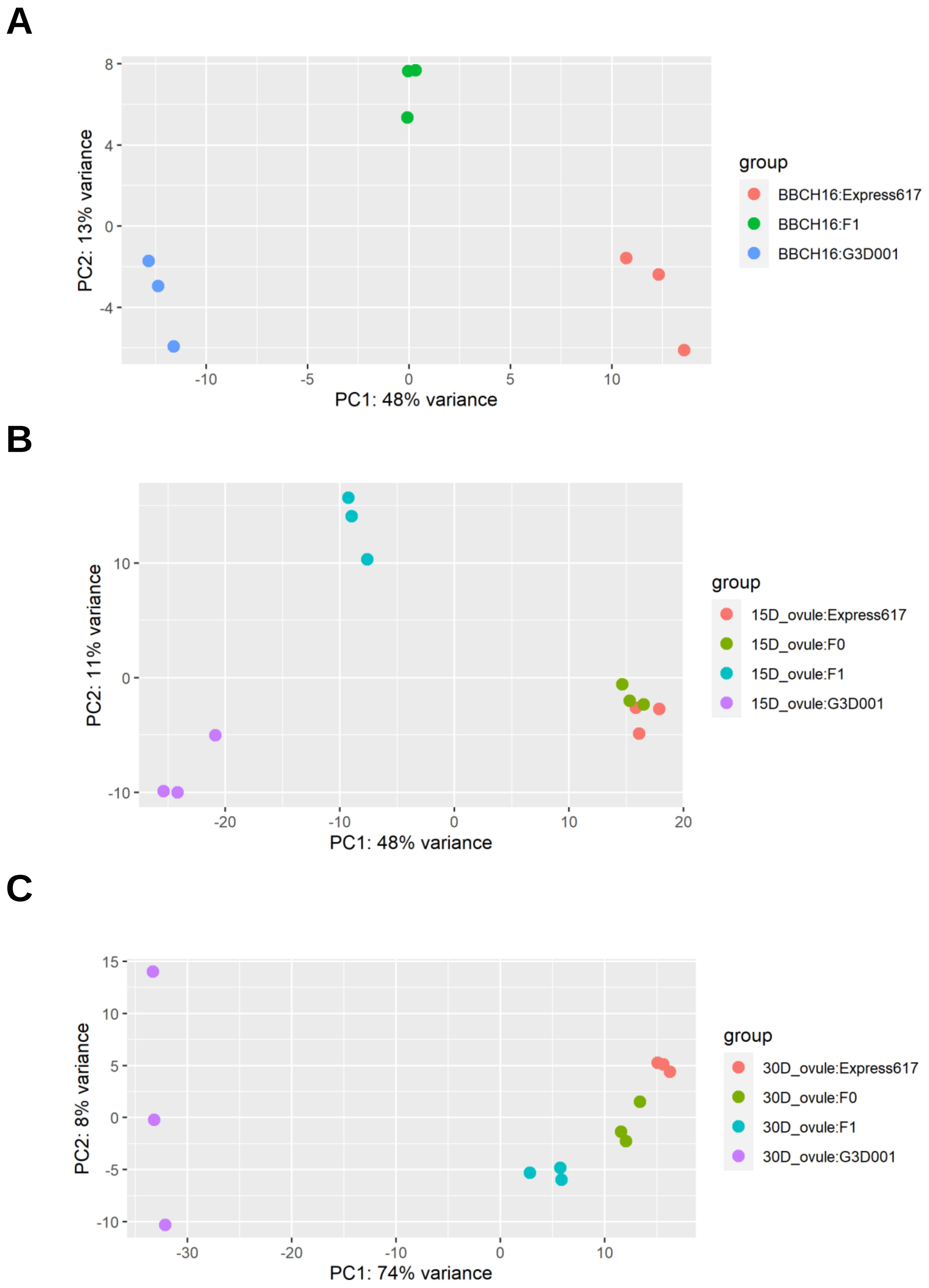

Supplementary Figure 3 Principal component analysis (PCA) of miRNA counts derived from sRNA-Seq libraries. Each circle represents a biological replicate during **(a)** the seedling, **(b)** 15 days after pollination and **(c)** 30 days after pollination stages.

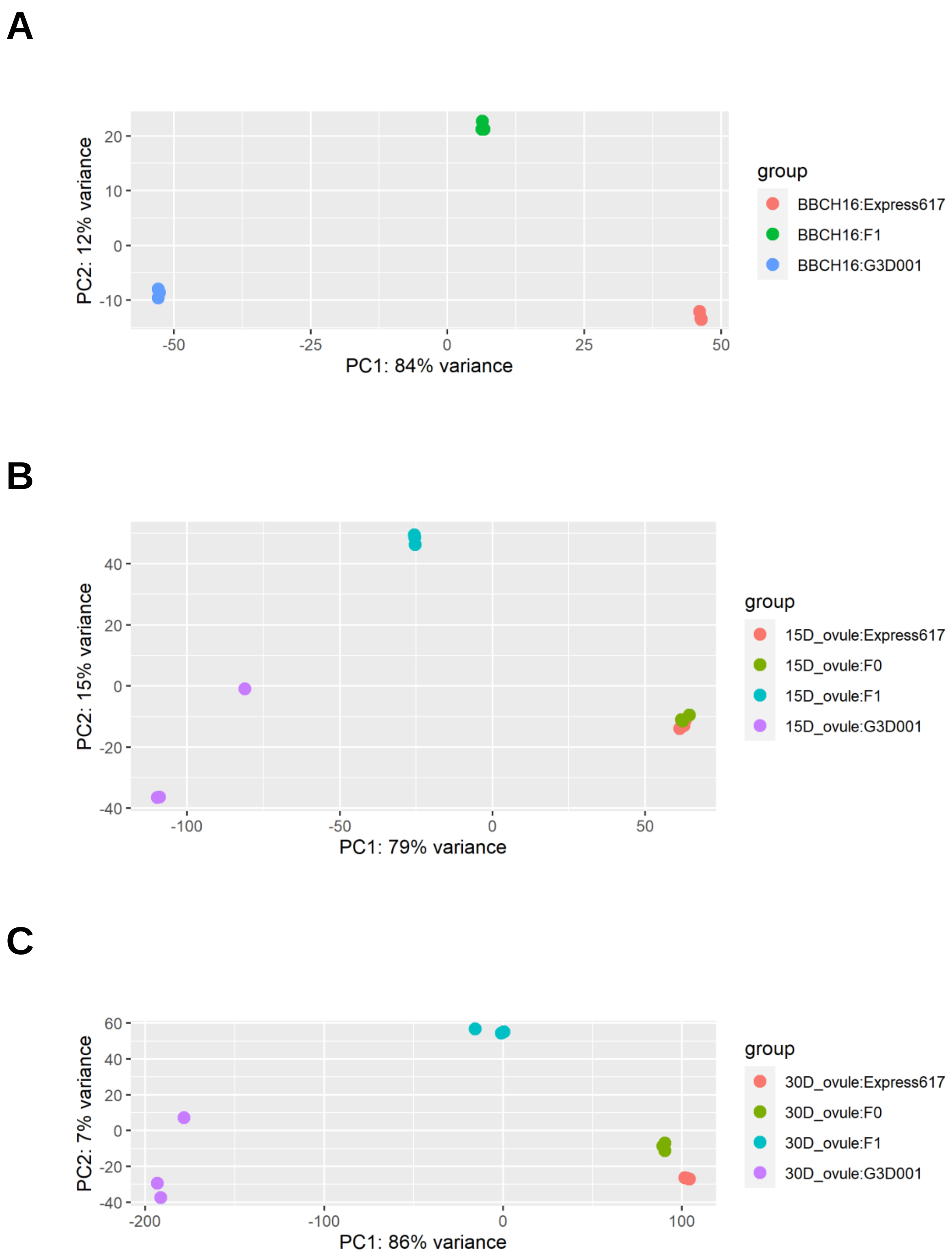

Supplementary Figure 4 Principal component analysis (PCA) of siRNA counts derived from sRNA-Seq libraries. Each circle represents a biological replicate during **(a)** the seedling, **(b)** 15 days after pollination and **(c)** 30 days after pollination stages.

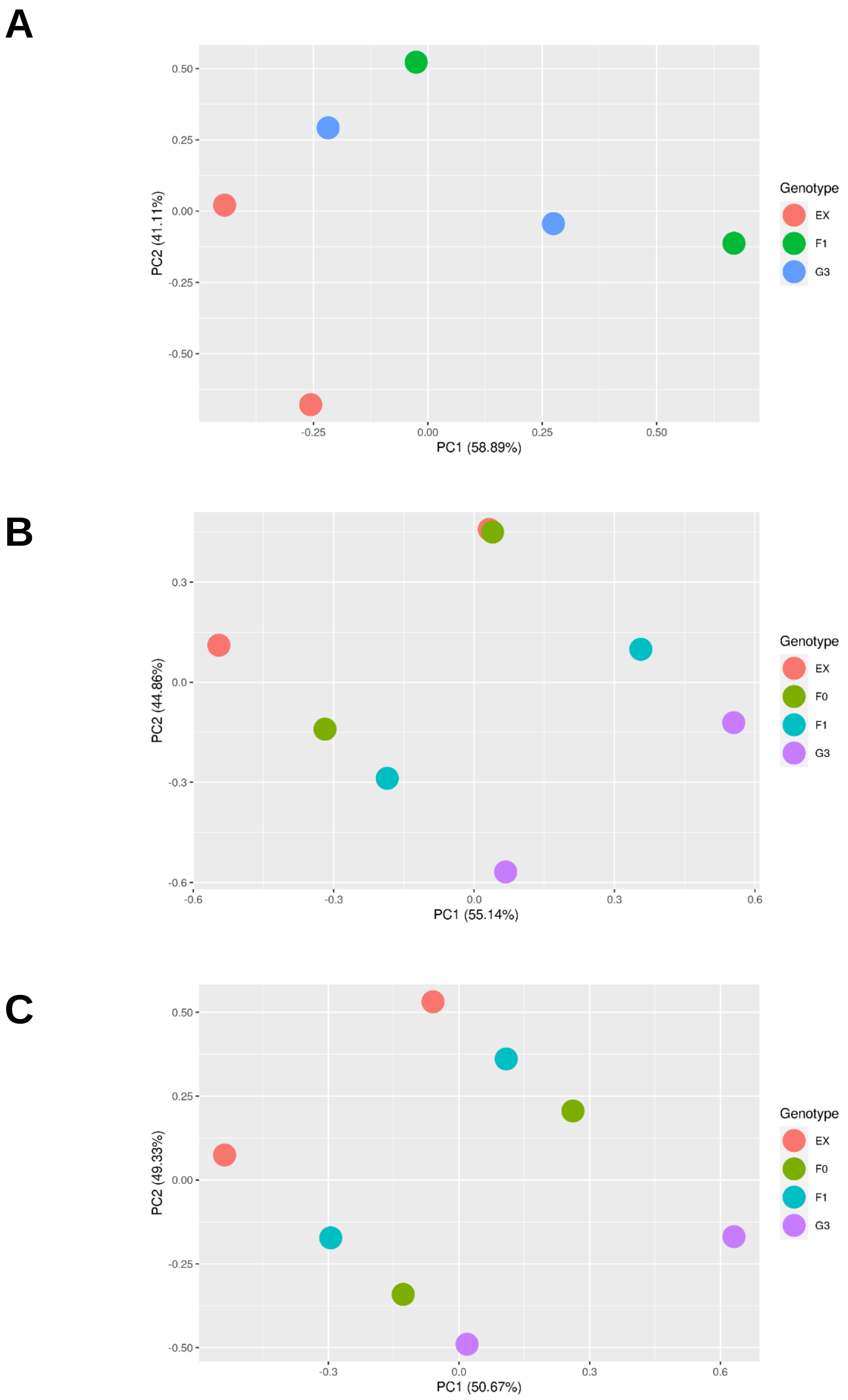

Supplementary Figure 5 Principal component analysis (PCA) of genome-wide CpG methylation levels derived from WGBS libraries. Each circle represents a biological replicate during **(a)** the seedling, **(b)** 15 days after pollination and **(c)** 30 days after pollination stages.

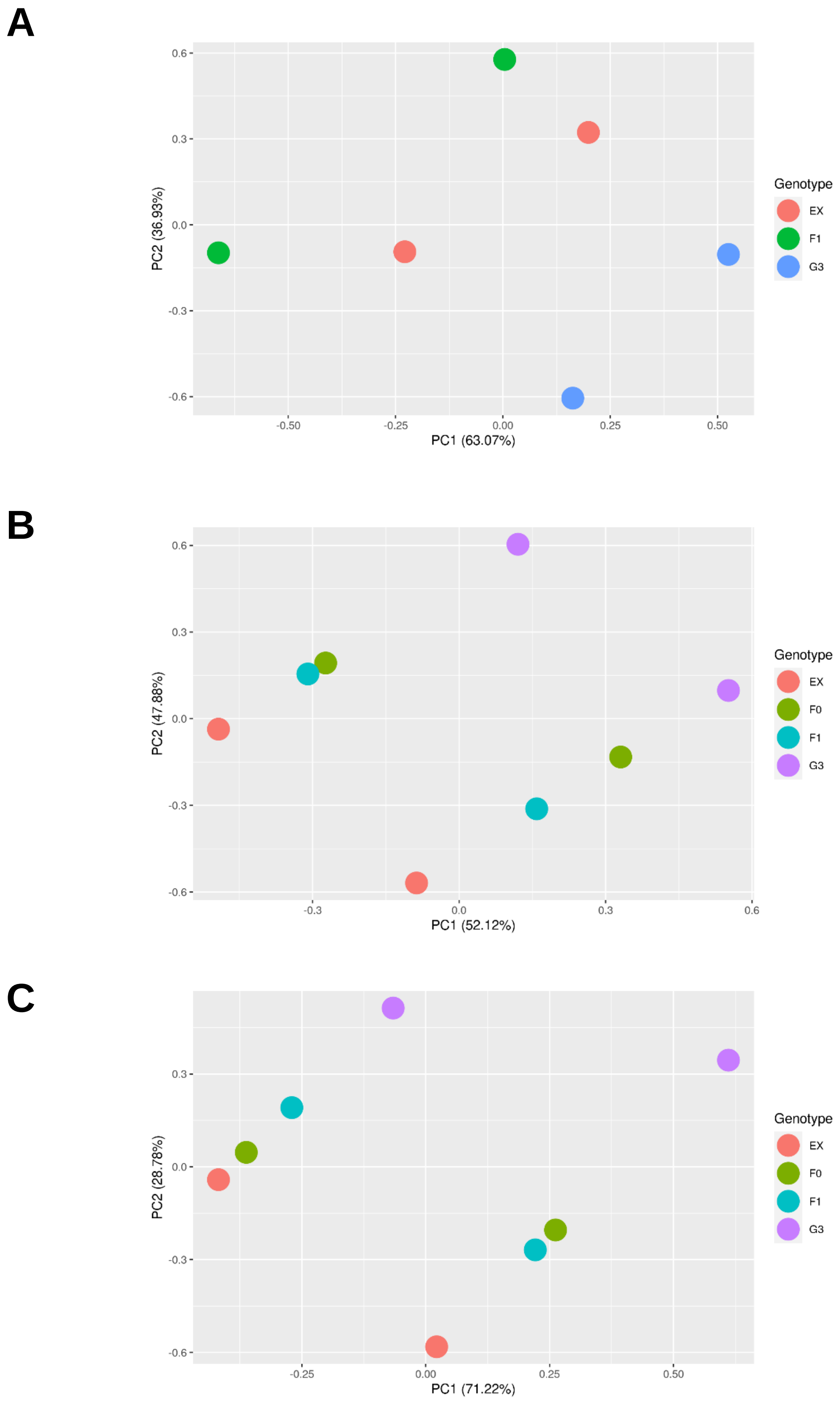

Supplementary Figure 6 Principal component analysis (PCA) of genome-wide CHG methylation levels derived from WGBS libraries. Each circle represents a biological replicate during **(a)** the seedling, (**b)** 15 days after pollination and **(c)** 30 days after pollination stages.

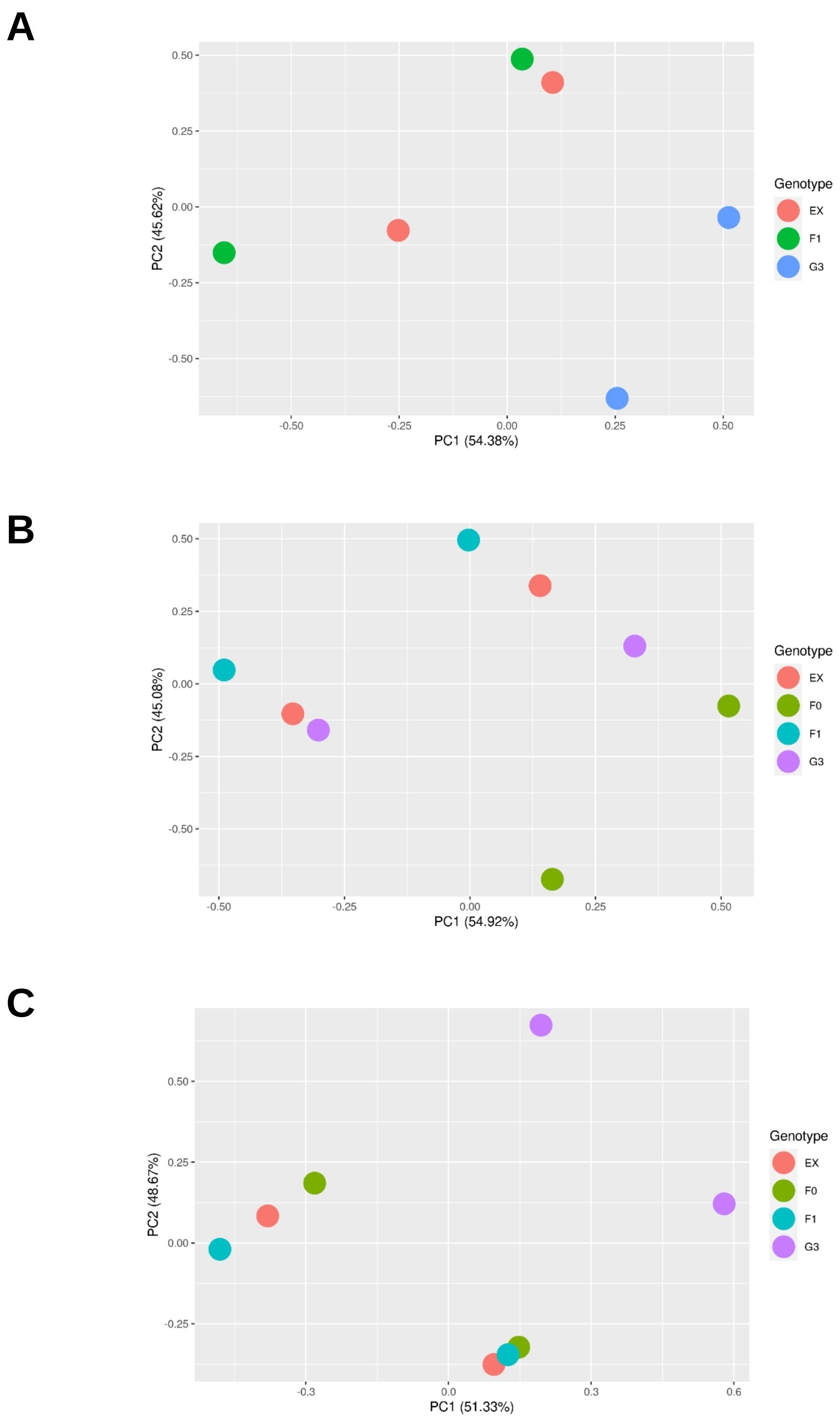

Supplementary Figure 7 Principal component analysis (PCA) of genome-wide CHH methylation levels derived from WGBS libraries. Each circle represents a biological replicate during **(a)** the seedling, (**b)** 15 days after pollination and **(c)** 30 days after pollination stages.

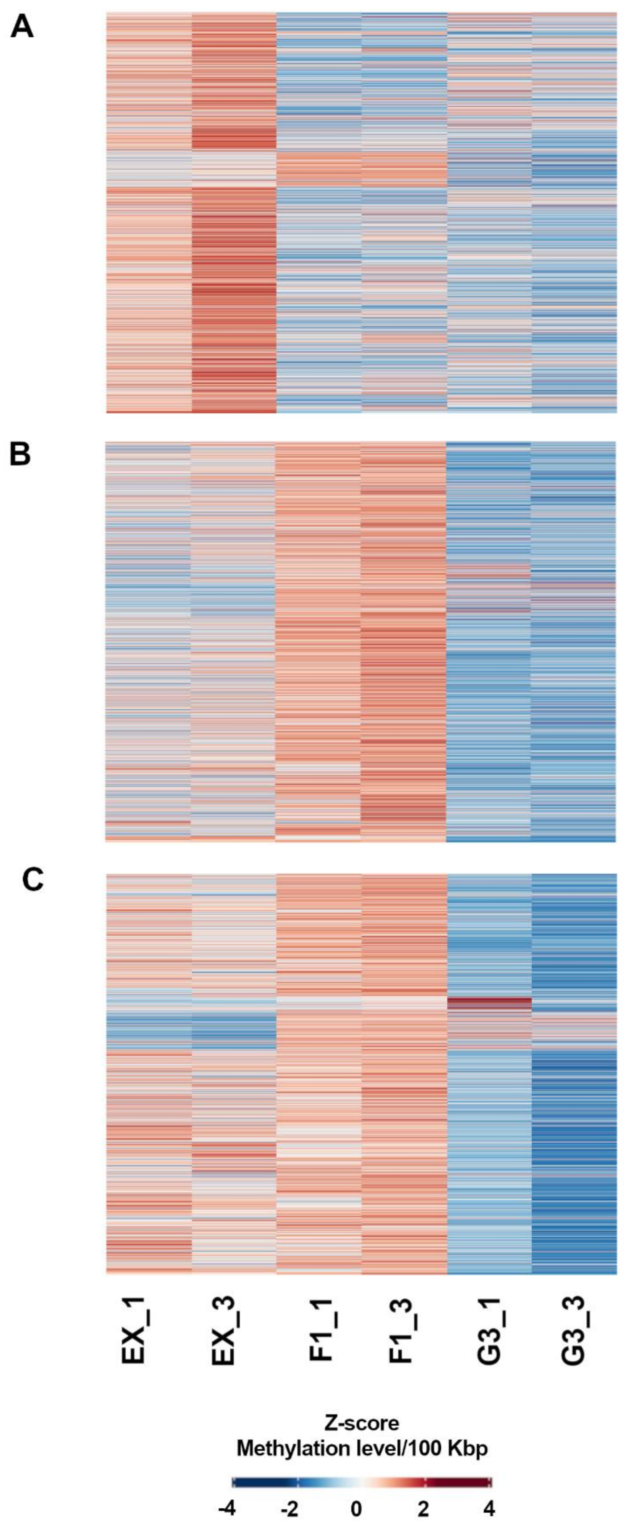

Supplementary Figure 8 Genome-wide methylation levels per 100 kbp bins from WGBS libraries during the seedling stage for the **(a)** CpG, **(b)** CHG and **(c)** CHH methylation contexts. Methylation levels were normalized and represented as Z-scores where higher methylation levels are in red and lower ones in blue**.** Each track represents a biological replicate from the Express617 (EX), G3D001 (G3) and F1 (F1) genotypes.

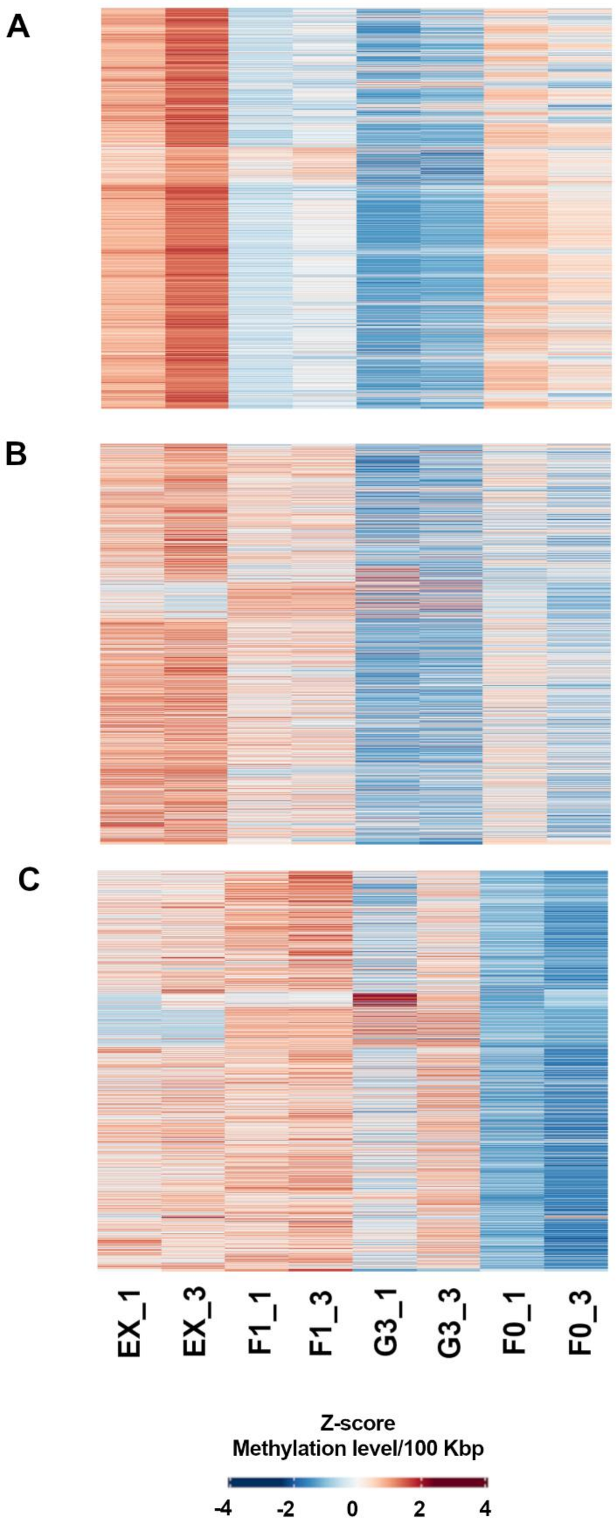

Supplementary Figure 9 Genome-wide methylation levels per 100 kbp bins from WGBS libraries during the 15 days after pollination stage for the **(a)** CpG, **(b)** CHG and **(c)** CHH methylation contexts. Methylation levels were normalized and represented as Z-scores where higher methylation levels are in red and lower ones in blue**.** Each track represents a biological replicate from the Express617 (EX), G3D001 (G3), F1 (F1) and F0 (F0) genotypes.

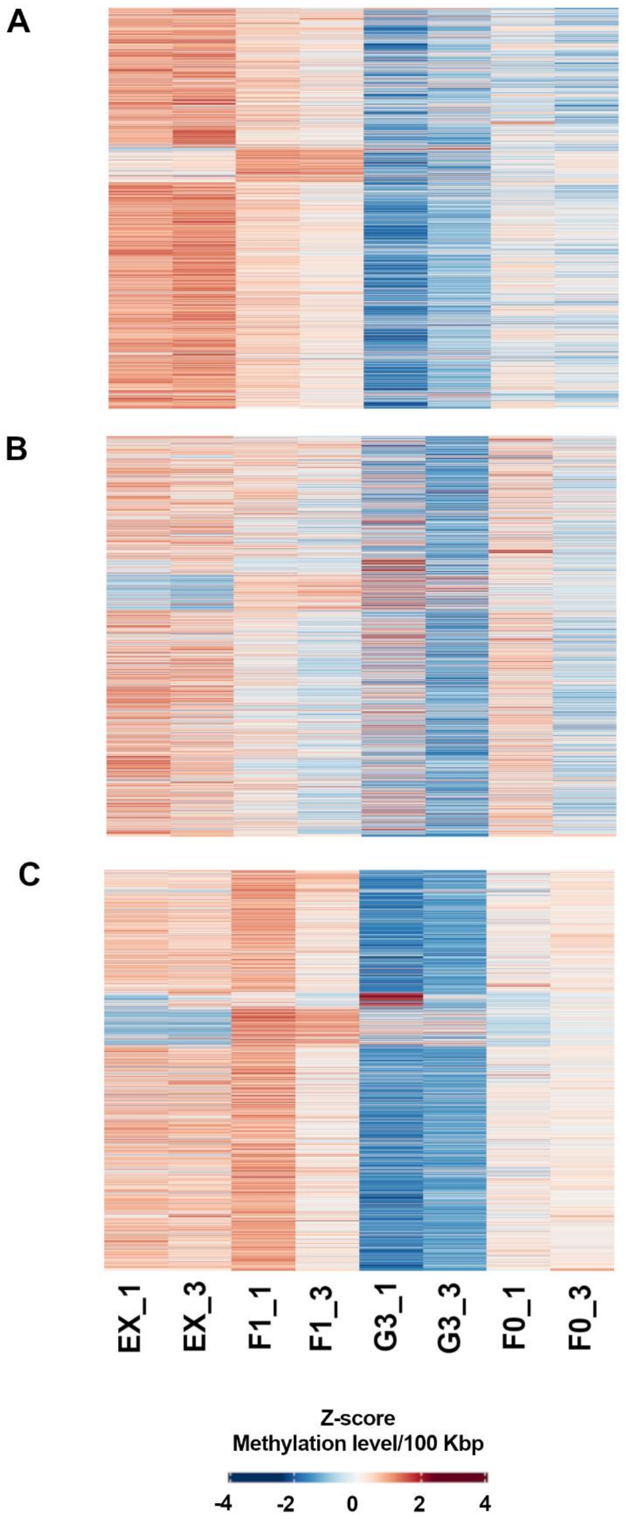

Supplementary Figure 10 Genome-wide methylation levels per 100 kbp bins from WGBS libraries during the 30 days after pollination stage for the **(a)** CpG, **(b)** CHG and **(c)** CHH methylation contexts. Methylation levels were normalized and represented as Z-scores where higher methylation levels are in red and lower ones in blue**.** Each track represents a biological replicate from the Express617 (EX), G3D001 (G3), F1 (F1) and F0 (F0) genotypes.

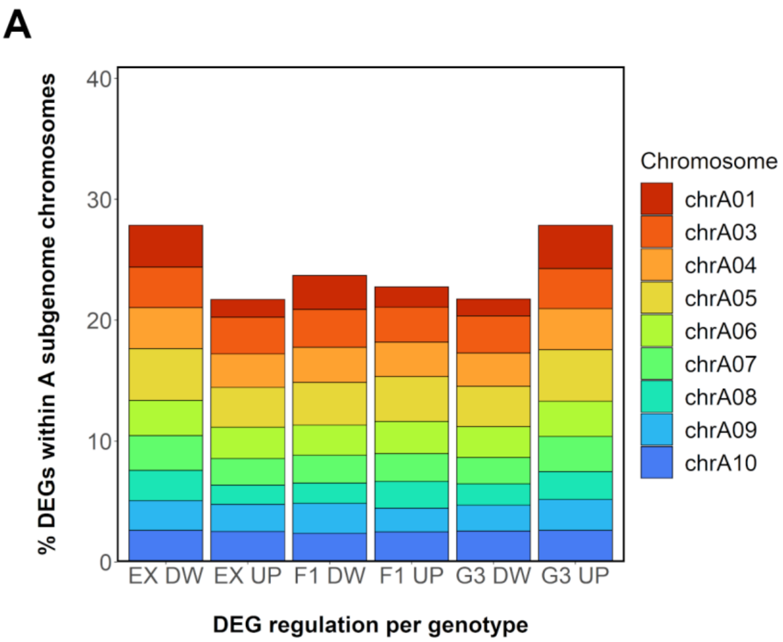

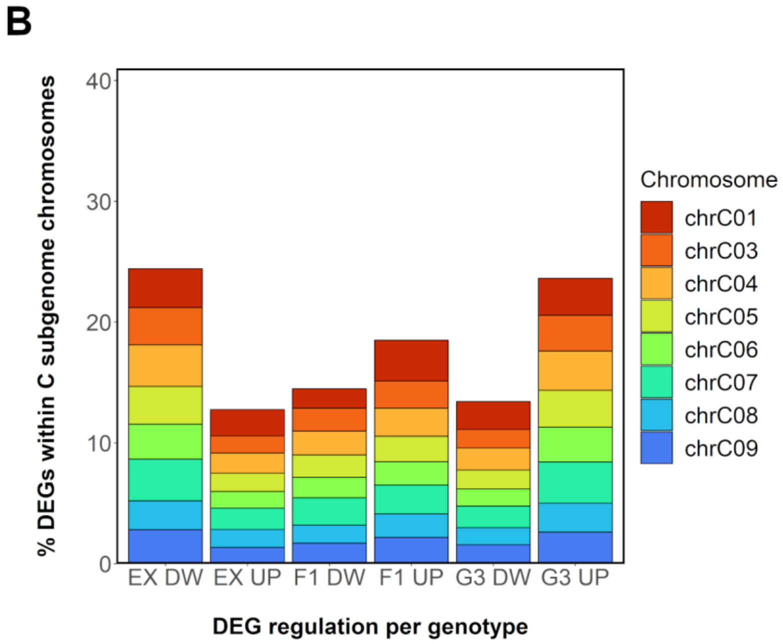

Supplementary Figure 11 Percentage of expressed upregulated and downregulated differentially expressed genes (DEGs) per subgenome and genotype during seedling stage (BBCH16). **a** Percentages of DEGs in respect to all genes per chromosome in A subgenome. **b** Percentages of DEGs in respect to all genes per chromosome in C subgenome. DEGs in genotypes (EX: Express 617, G3: G3D001 and F1) are classified either as upregulated (UP) or downregulated (DW). Percentages are calculated based on the number of DEGs observed in each subgenome. Chromosomes A02 and C02 were excluded to discard analysis bias due to whole A02 chromosome duplication and whole chromosome C02 deletion observed in G3D001 (Orantes-Bonilla et al., 2022).

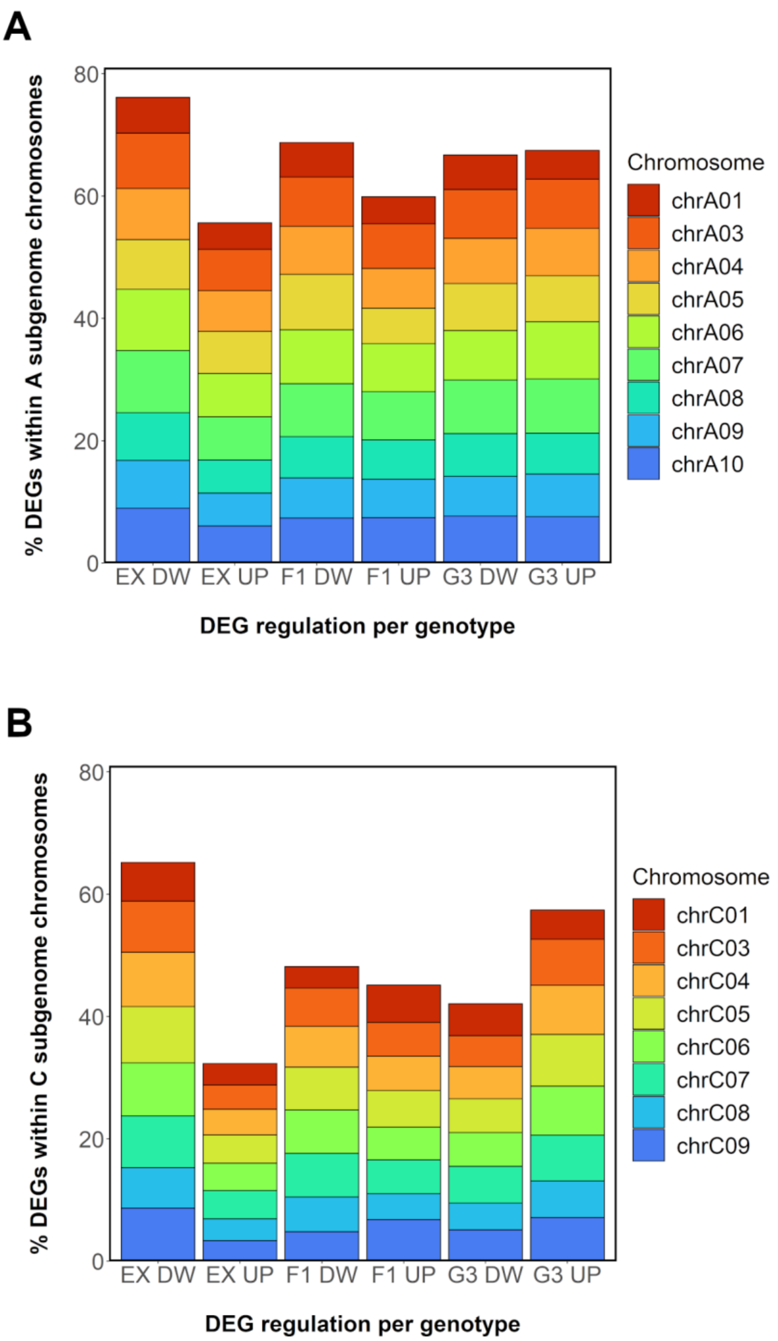

Supplementary Figure 12 Percentage of expressed upregulated and downregulated differentially expressed genes (DEGs) per subgenome and genotype in 15 days after pollination ovules in F1 and parents. **a** Percentages of DEGs in respect to all genes per chromosome in A subgenome. **b** Percentages of DEGs in respect to all genes per chromosome in C subgenome. DEGs in genotypes (EX: Express 617, G3: G3D001 and F1) are classified either as upregulated (UP) or downregulated (DW). Percentages are calculated based on the number of DEGs observed in each subgenome. Chromosomes A02 and C02 were excluded to discard analysis bias due to whole A02 chromosome duplication and whole chromosome C02 deletion observed in G3D001 (Orantes-Bonilla et al., 2022).

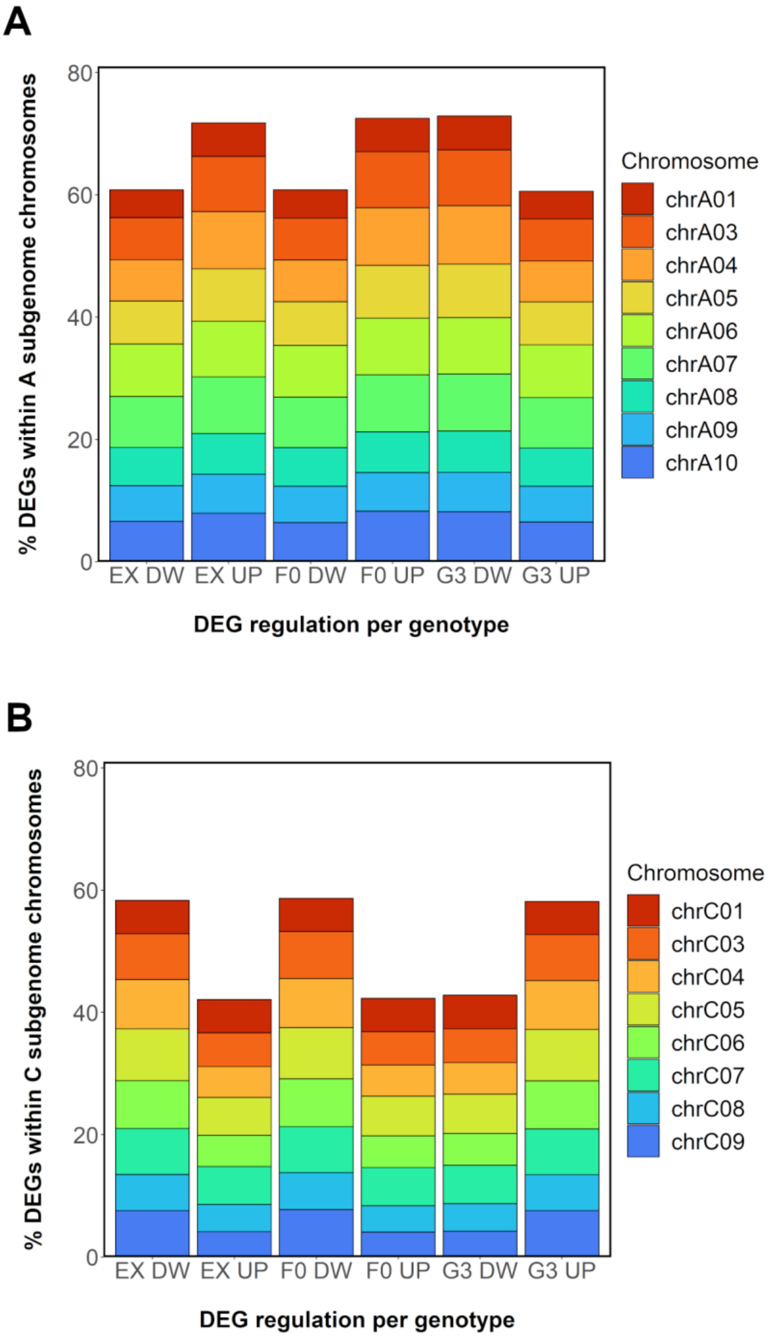

Supplementary Figure 13 Percentage of expressed upregulated and downregulated differentially expressed genes (DEGs) per subgenome and genotype in 15 days after pollination ovules in F0 and parents. **a** Percentages of DEGs in respect to all genes per chromosome in A subgenome. **b** Percentages of DEGs in respect to all genes per chromosome in C subgenome. DEGs in genotypes (EX: Express 617, G3: G3D001 and F0) are classified either as upregulated (UP) or downregulated (DW). Percentages are calculated based on the number of DEGs observed in each subgenome. Chromosomes A02 and C02 were excluded to discard analysis bias due to whole A02 chromosome duplication and whole chromosome C02 deletion observed in G3D001 (Orantes-Bonilla et al., 2022).

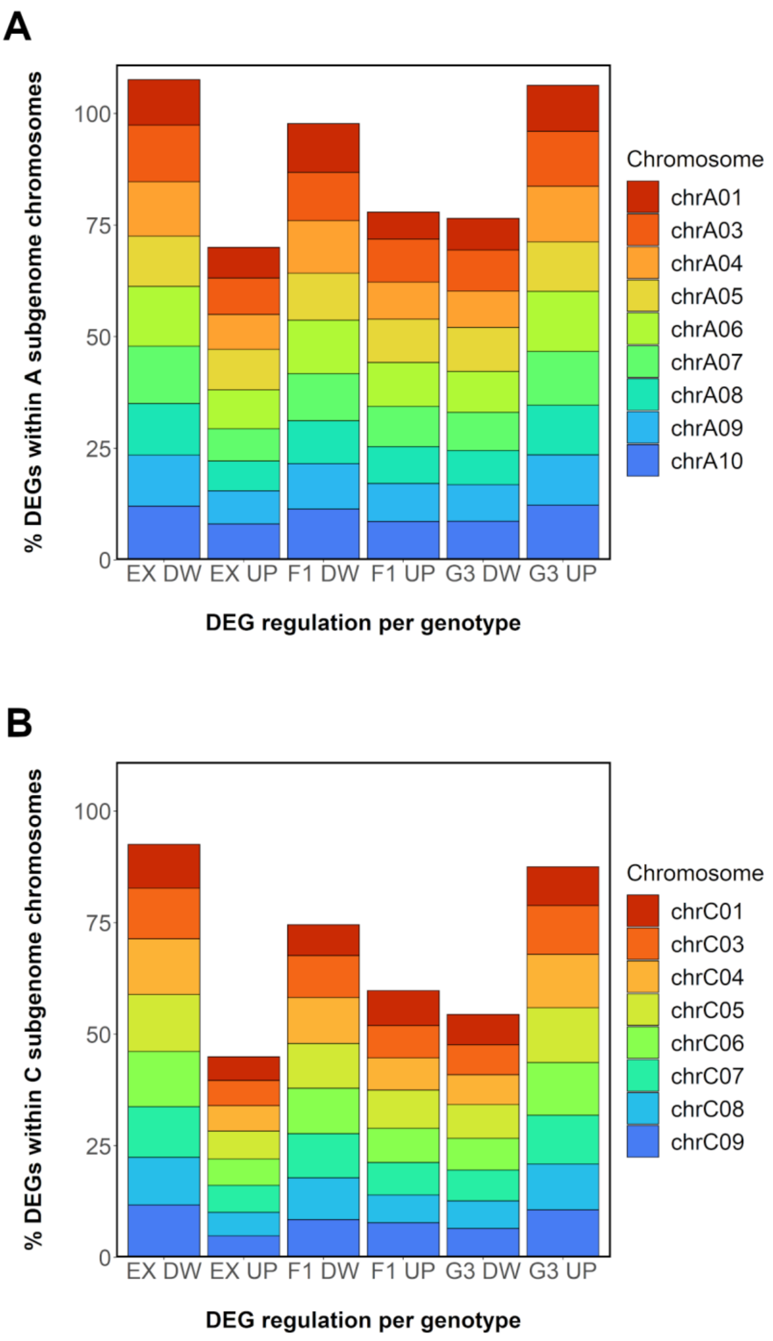

Supplementary Figure 14 Percentage of expressed upregulated and downregulated differentially expressed genes (DEGs) per subgenome and genotype in 30 days after pollination ovules in F1 and parents. **a** Percentages of DEGs in respect to all genes per chromosome in A subgenome. **b** Percentages of DEGs in respect to all genes per chromosome in C subgenome. DEGs in genotypes (EX: Express 617, G3: G3D001 and F1) are classified either as upregulated (UP) or downregulated (DW). Percentages are calculated based on the number of DEGs observed in each subgenome. Chromosomes A02 and C02 were excluded to discard analysis bias due to whole A02 chromosome duplication and whole chromosome C02 deletion observed in G3D001 (Orantes-Bonilla et al., 2022).

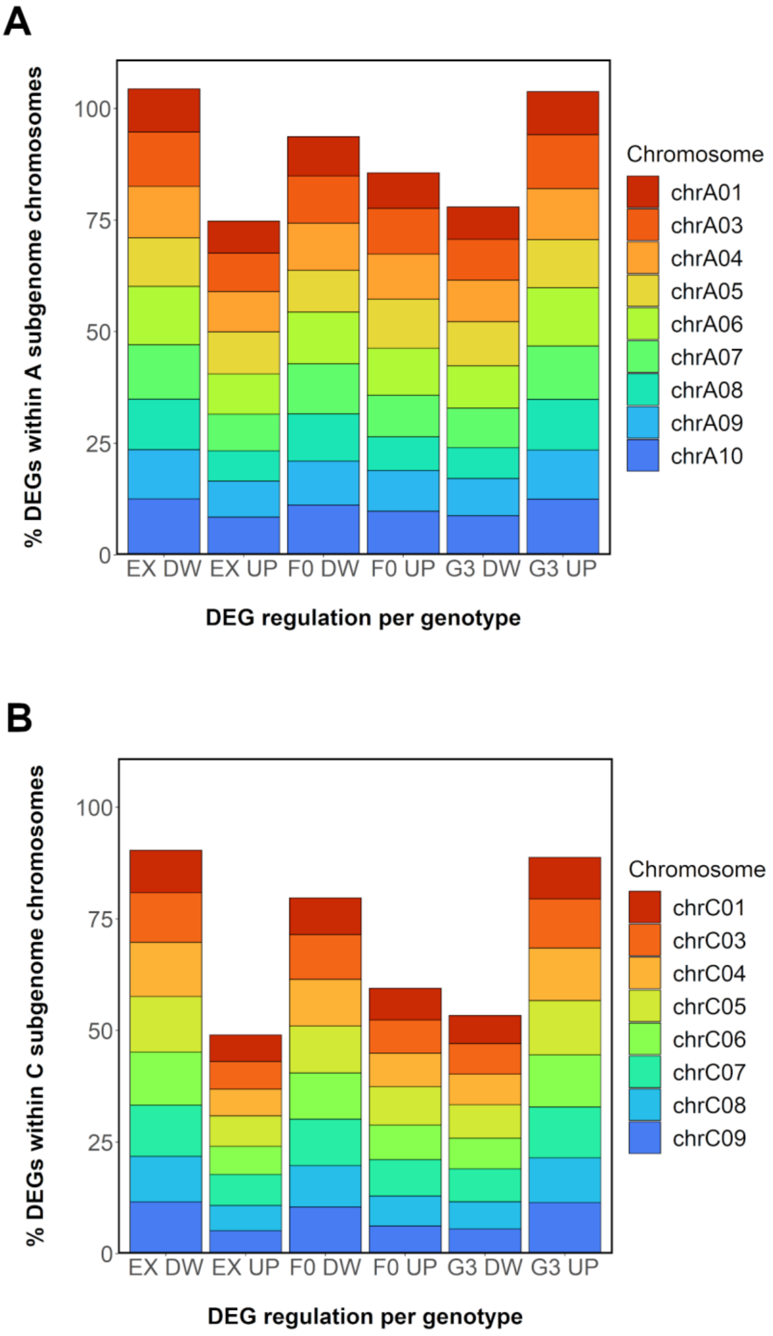

Supplementary Figure 15 Percentage of expressed upregulated and downregulated differentially expressed genes (DEGs) per subgenome and genotype in 30 days after pollination ovules in F0 and parents. **a** Percentages of DEGs in respect to all genes per chromosome in A subgenome. **b** Percentages of DEGs in respect to all genes per chromosome in C subgenome. DEGs in genotypes (EX: Express 617, G3: G3D001 and F0) are classified either as upregulated (UP) or downregulated (DW). Percentages are calculated based on the number of DEGs observed in each subgenome. Chromosomes A02 and C02 were excluded to discard analysis bias due to whole A02 chromosome duplication and whole chromosome C02 deletion observed in G3D001 (Orantes-Bonilla et al., 2022).

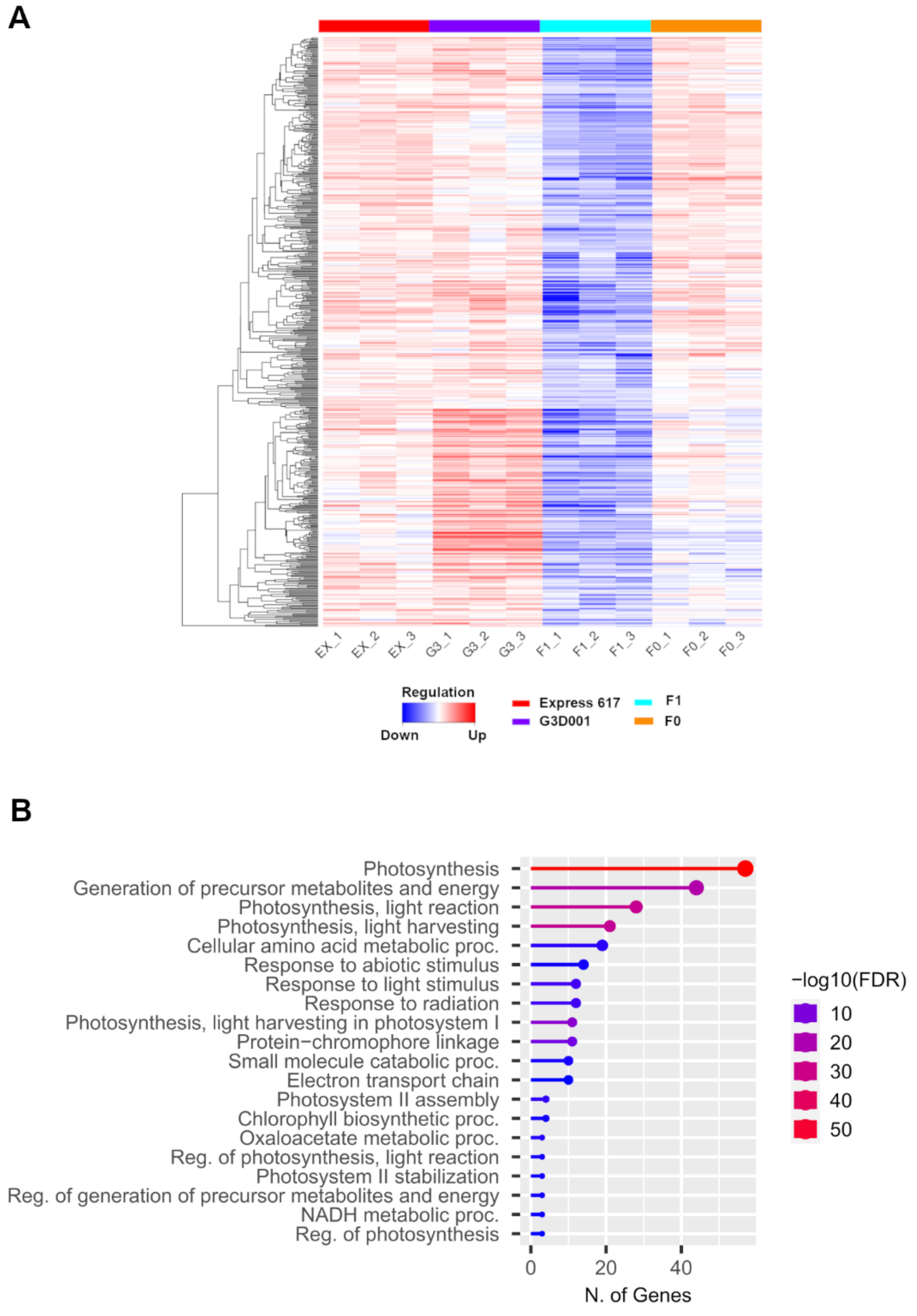

Supplementary Figure 16 (**a**) Gene expression heatmap and (**b**) gene ontology (GO) enrichment of biological processes from 15 days after pollination ovules with transgressive downregulation patterns in the F1.

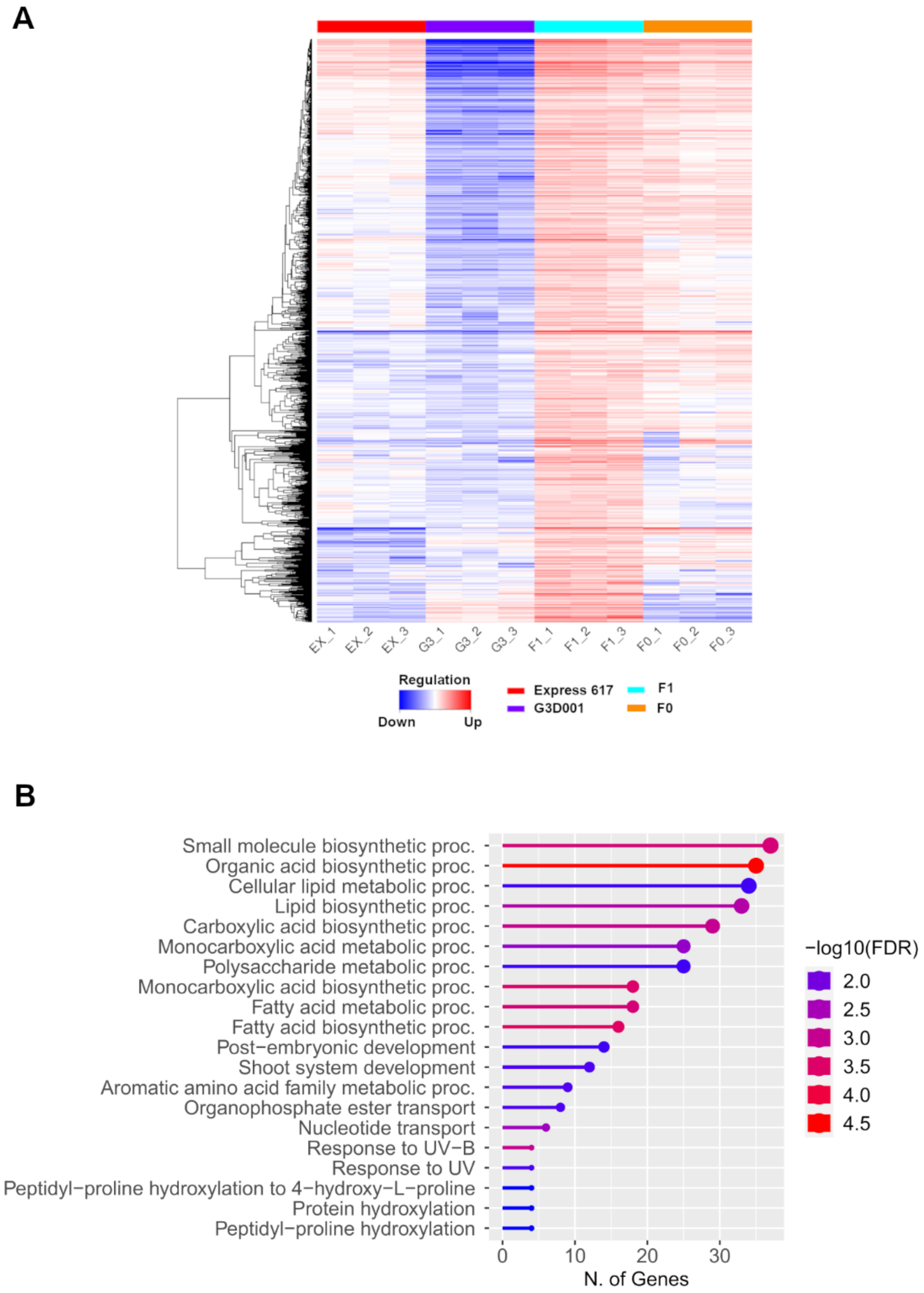

Supplementary Figure 17 (**a**) Gene expression heatmap and (**b**) gene ontology (GO) enrichment of biological processes from 30 days after pollination ovules with transgressive upregulation patterns in the F1**.**

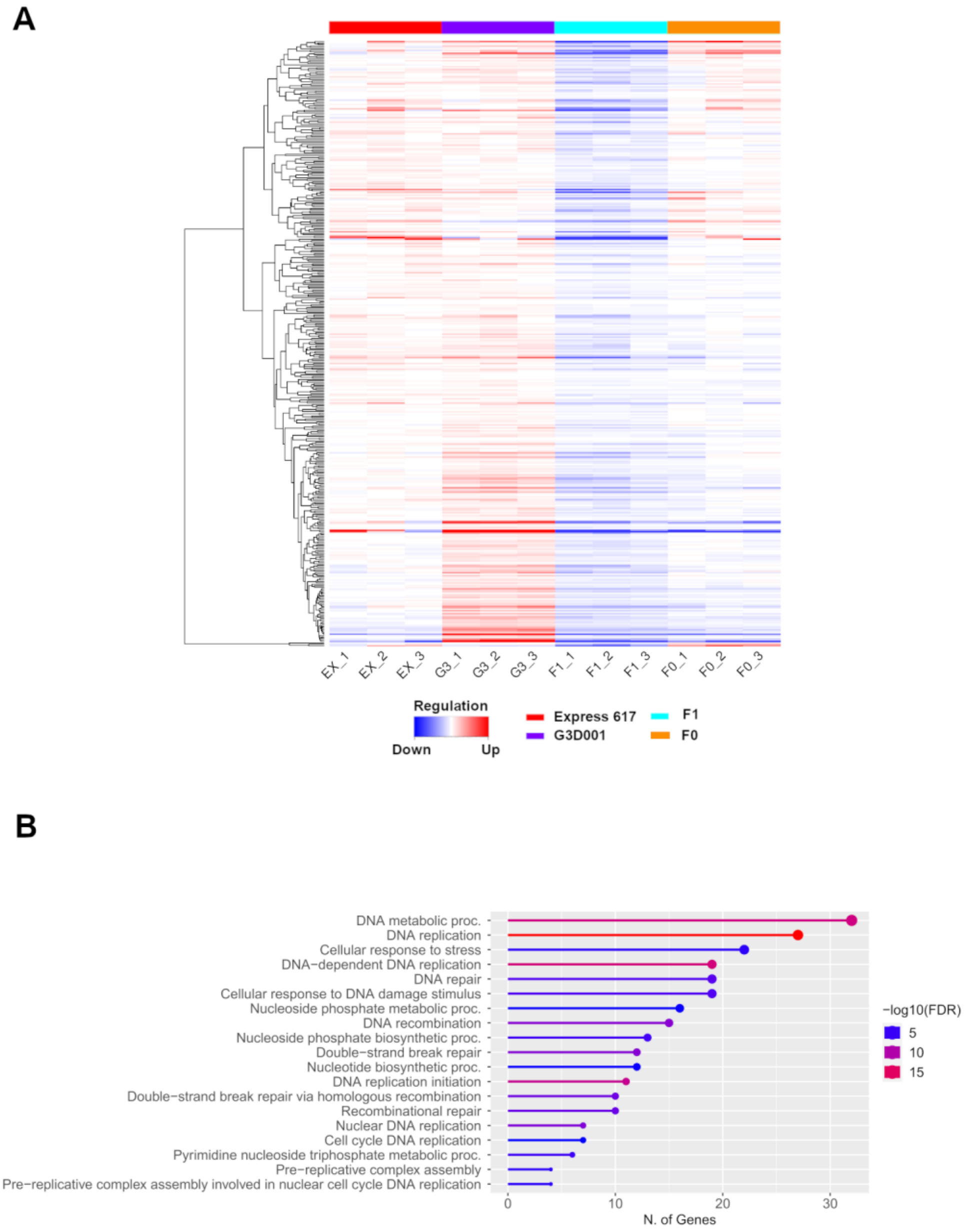

Supplementary Figure 18 (**a**) Gene expression heatmap and (**b**) gene ontology (GO) enrichment of biological processes from 30 days after pollination ovules with transgressive downregulation patterns in the F1.

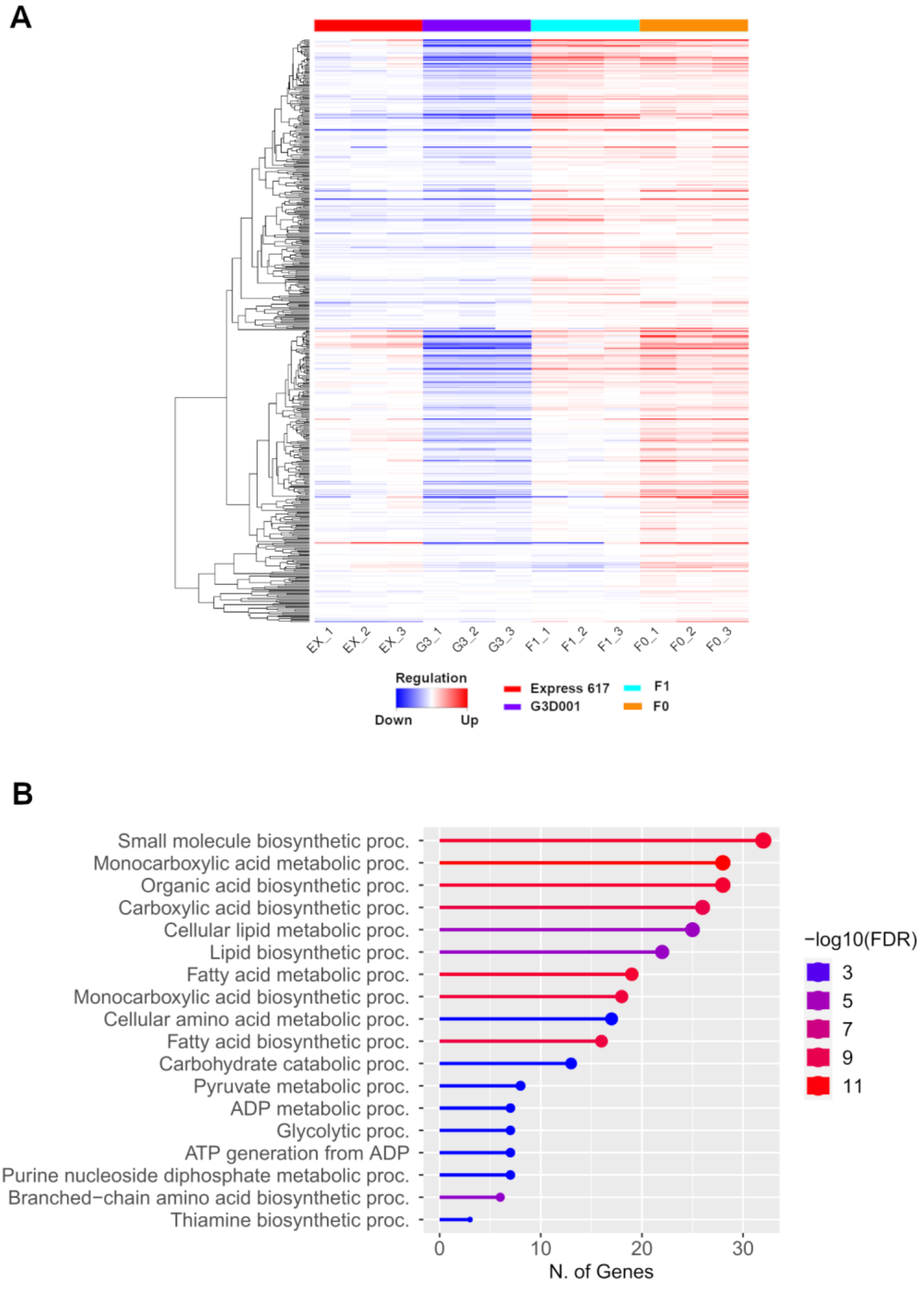

Supplementary Figure 19 (**a**) Gene expression heatmap and (**b**) gene ontology (GO) enrichment of biological processes from 30 days after pollination ovules with transgressive upregulation patterns in the F0.

A

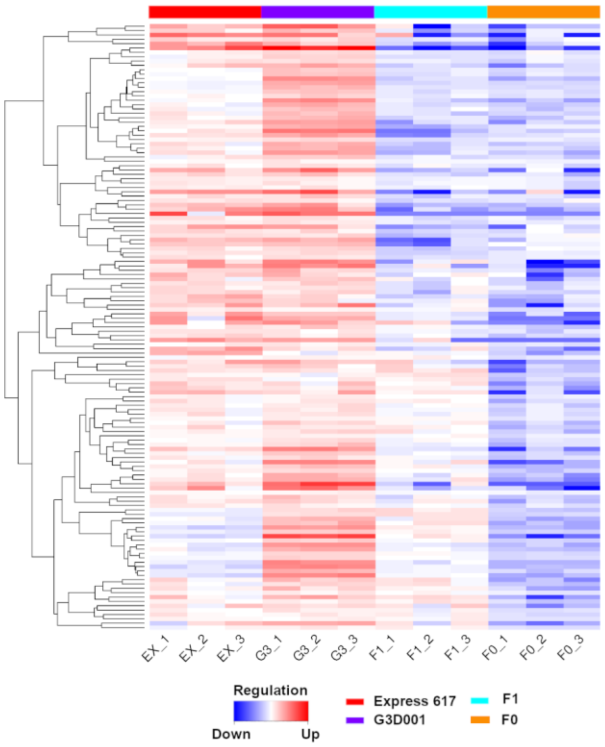

B

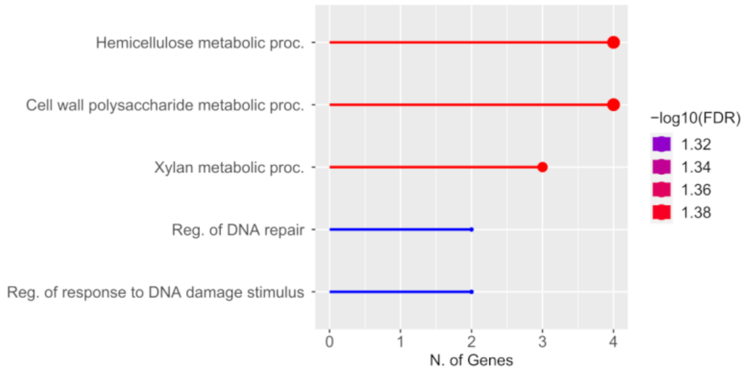

Supplementary Figure 20 (**a**) Gene expression heatmap and (**b**) gene ontology (GO) enrichment of biological processes from 30 days after pollination ovules with transgressive upregulation patterns in the F0.

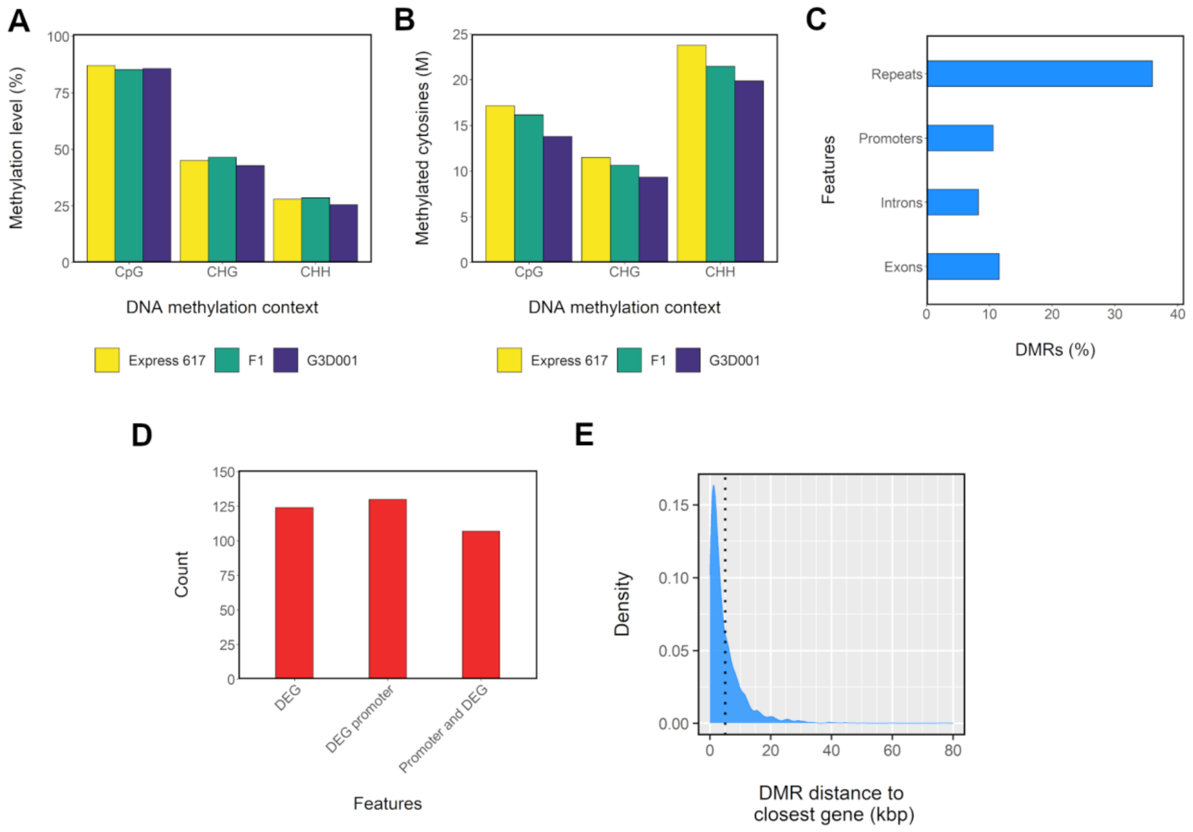

Supplementary Figure 21 Methylation patterns in seedling stage (BBCH16) from F1 and parents. **a** Methylation level per genotype and DNA methylation context. **b** Count of methylated cytosines in million (M) scale per genotype and DNA methylation context. **c** Distribution of differentially methylated regions (DMRs) across introns, exons, repeats and promoters (1 kbp upstream from gene start). **d** Distribution of methylated differential expressed genes (DEGs) and their promoters. **e** Kernel density estimation (KED)-based distribution of DMRs distance to closest gene. A dotted line is used to delimit DMRs located 5 kbp from a gene.

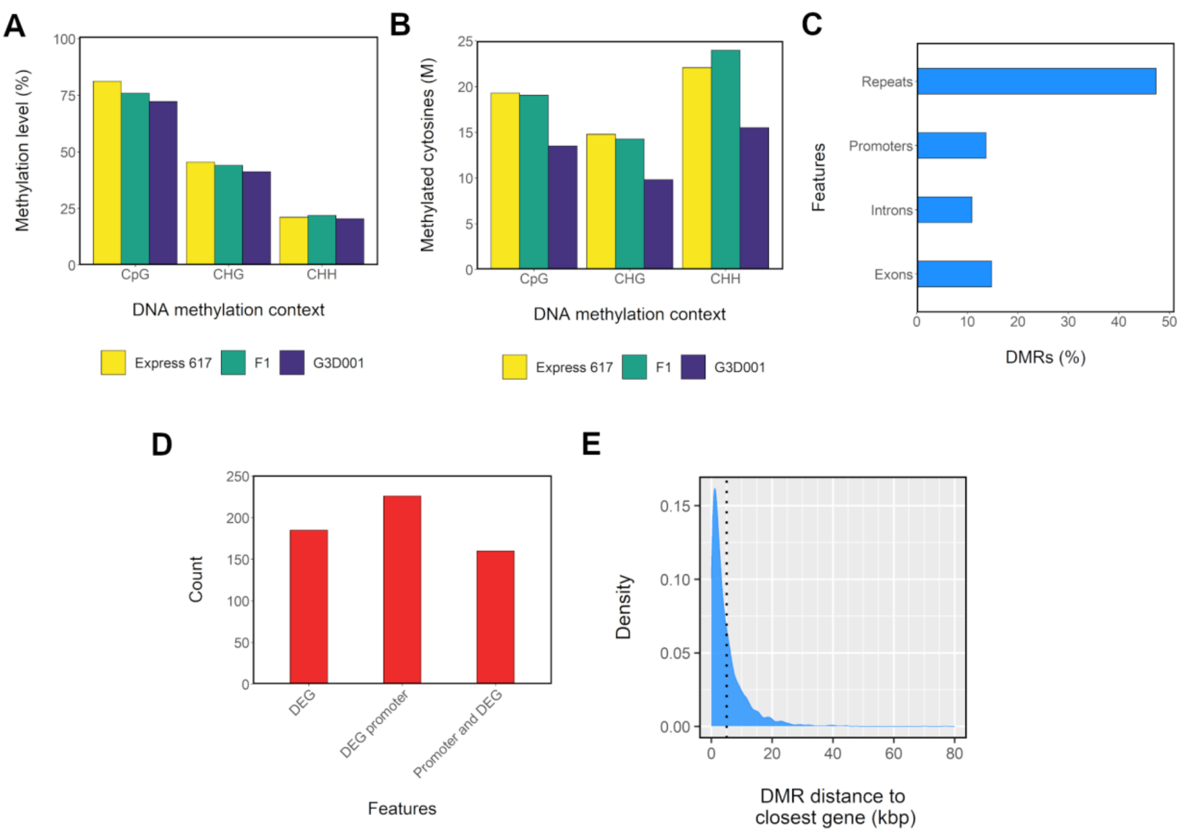

Supplementary Figure 22 Methylation patterns in 15 days after pollination ovules from F1 and parents. **a** Methylation level per genotype and DNA methylation context. **b** Count of methylated cytosines in million (M) scale per genotype and DNA methylation context. **c** Distribution of differentially methylated regions (DMRs) across introns, exons, repeats and promoters (1 kbp upstream from gene start). **d** Distribution of methylated differential expressed genes (DEGs) and their promoters. **e** Kernel density estimation (KED)-based distribution of DMRs distance to closest gene. A dotted line is used to delimit DMRs located 5 kbp from a gene.

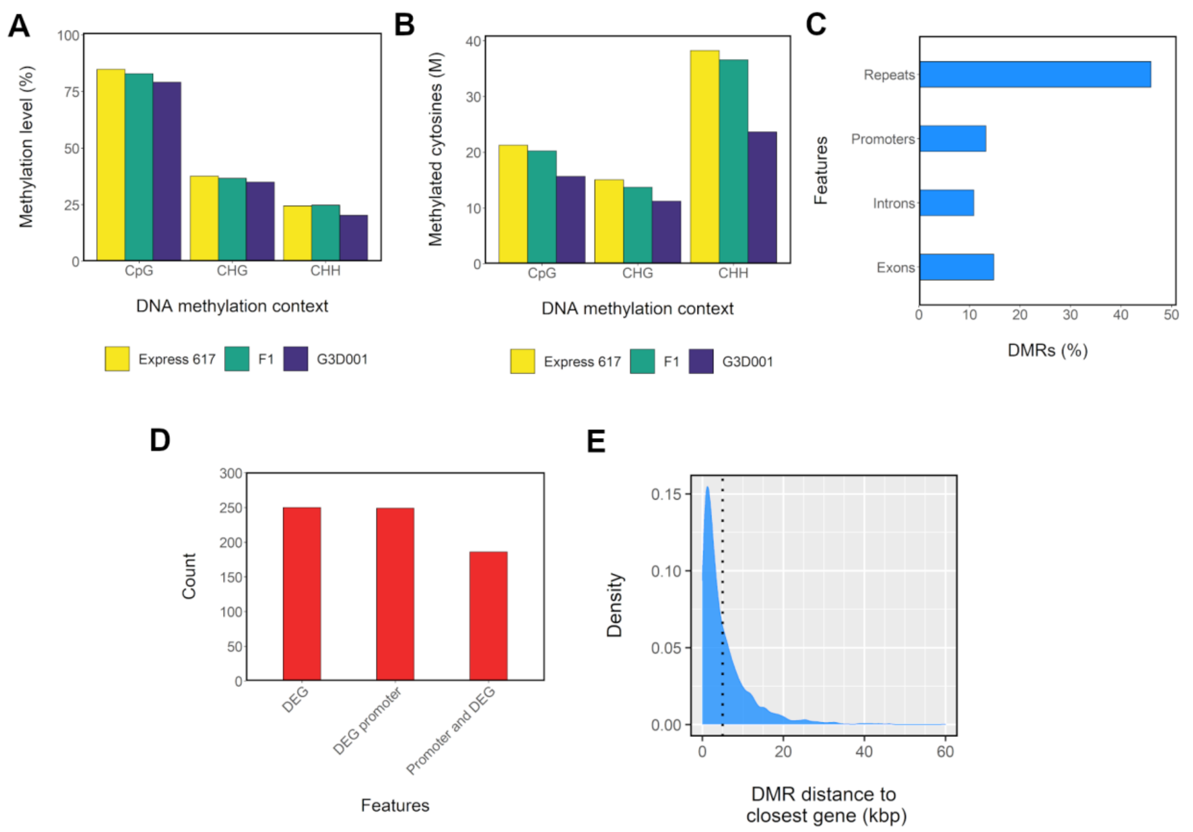

Supplementary Figure 23 Methylation patterns in 30 days after pollination ovules from F1 and parents. **a** Methylation level per genotype and DNA methylation context. **b** Count of methylated cytosines in million (M) scale per genotype and DNA methylation context. **c** Distribution of differentially methylated regions (DMRs) across introns, exons, repeats and promoters (1 kbp upstream from gene start). **d** Distribution of methylated differential expressed genes (DEGs) and their promoters. **e** Kernel density estimation (KED)-based distribution of DMRs distance to closest gene. A dotted line is used to delimit DMRs located 5 kbp from a gene.

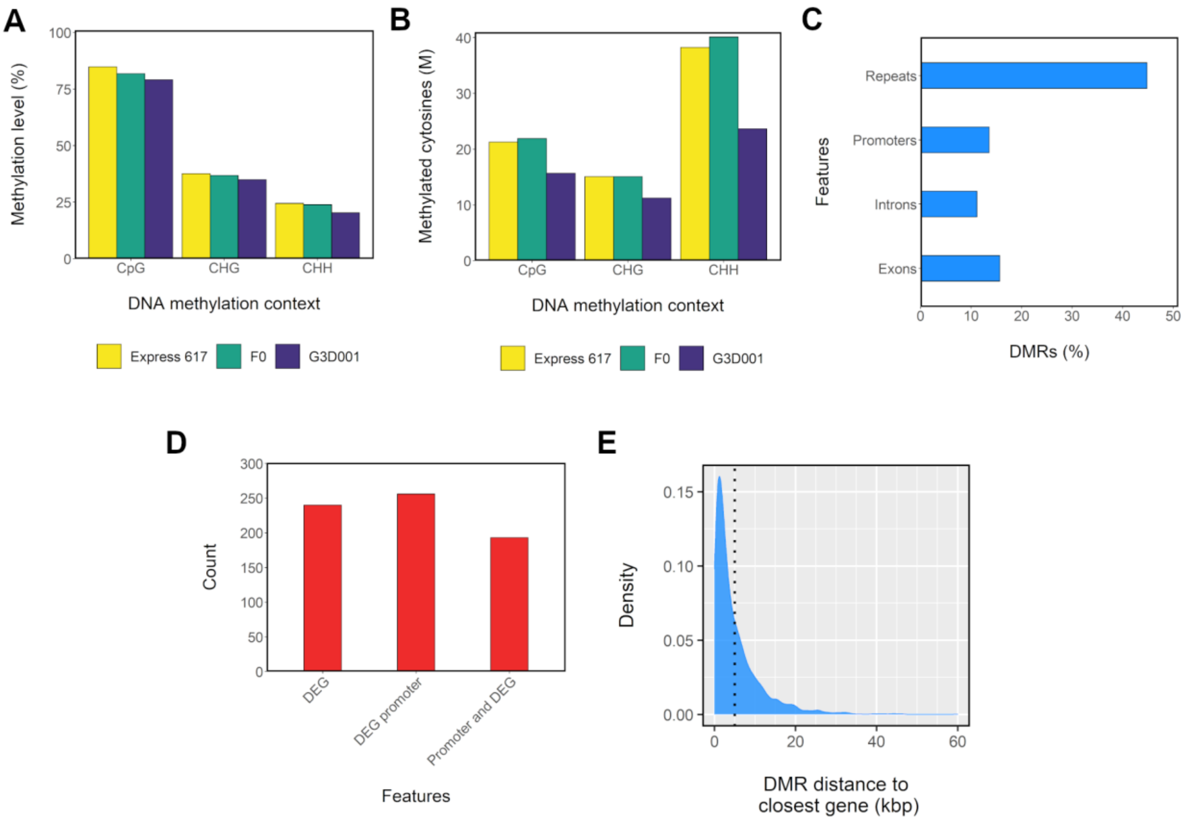

Supplementary Figure 24 Methylation patterns in 30 days after pollination ovules from F0 and parents. **a** Methylation level per genotype and DNA methylation context. **b** Count of methylated cytosines in million (M) scale per genotype and DNA methylation context. **c** Distribution of differentially methylated regions (DMRs) across introns, exons, repeats and promoters (1 kbp upstream from gene start). **d** Distribution of methylated differential expressed genes (DEGs) and their promoters. **e** Kernel density estimation (KED)-based distribution of DMRs distance to closest gene. A dotted line is used to delimit DMRs located 5 kbp from a gene.

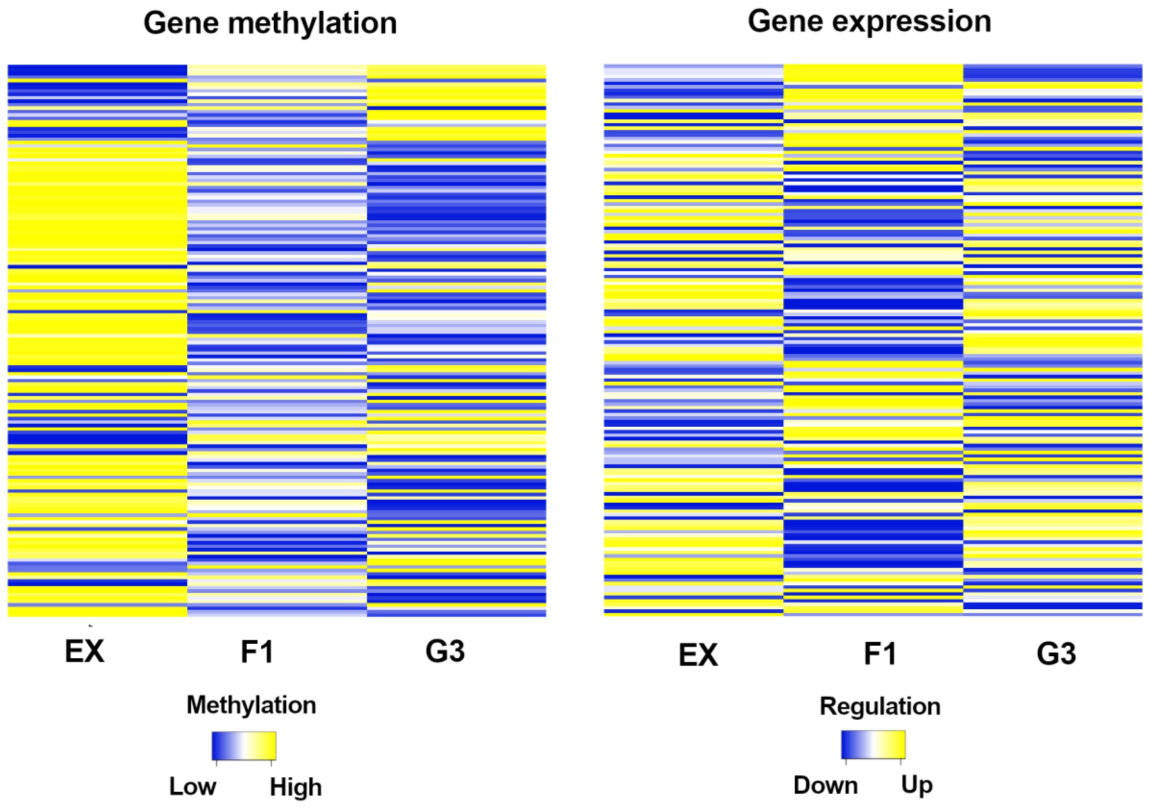

Supplementary Figure 25 Gene expression and gene body and promoter methylation in CpG and CHG contexts from 15 days after pollination ovules displaying transgressive patterns in the F1 and its parents. Genes are sorted in the same order in both heatmaps.

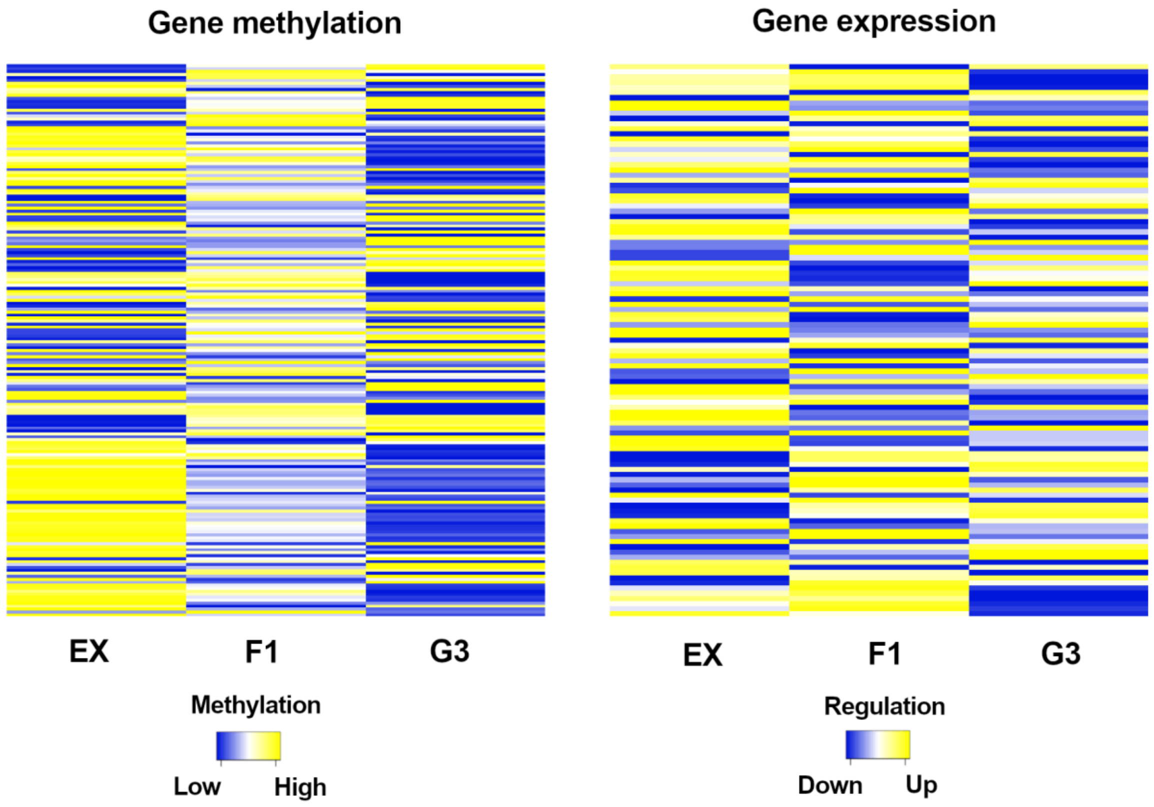

Supplementary Figure 26 Gene expression and gene body and promoter methylation in CpG and CHG contexts from 30 days after pollination ovules displaying transgressive patterns in the F1 and its parents. Genes are sorted in the same order in both heatmaps.

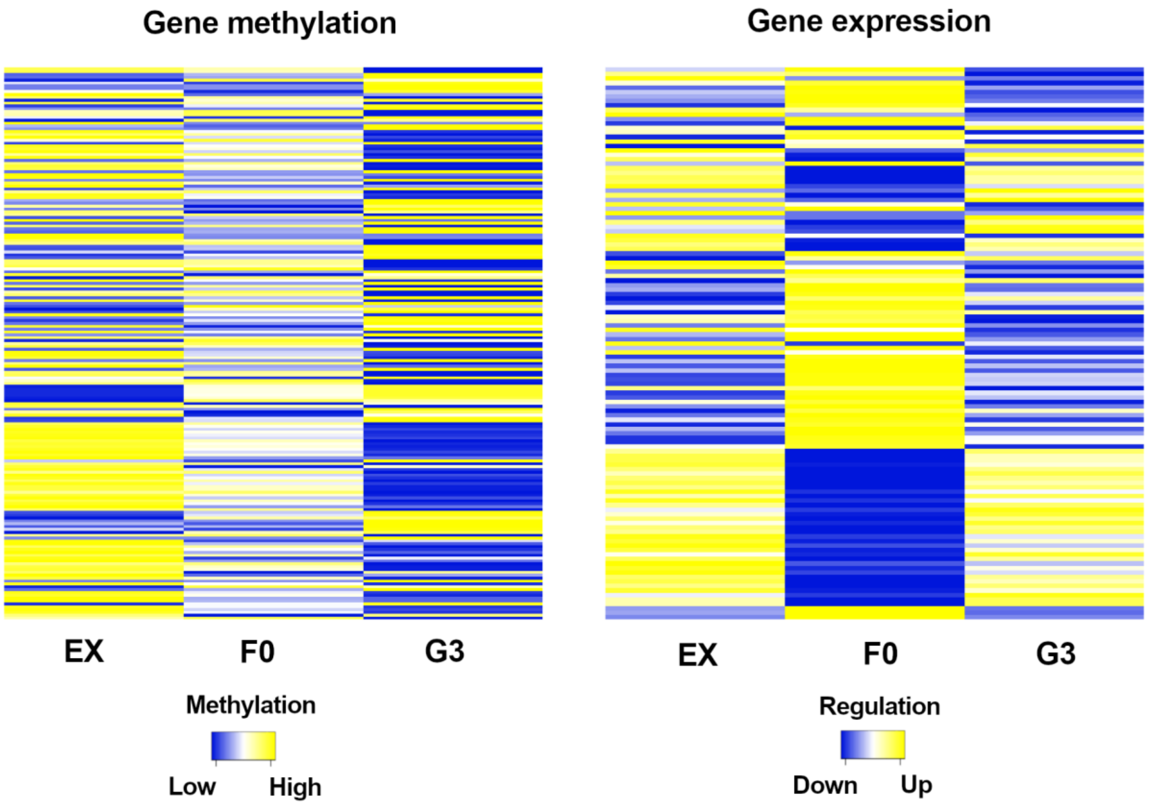

Supplementary Figure 27 Gene expression and gene body and promoter methylation in CpG and CHG contexts from 30 days after pollination ovules displaying transgressive patterns in the F0 and its parents. Genes are sorted in the same order in both heatmaps.

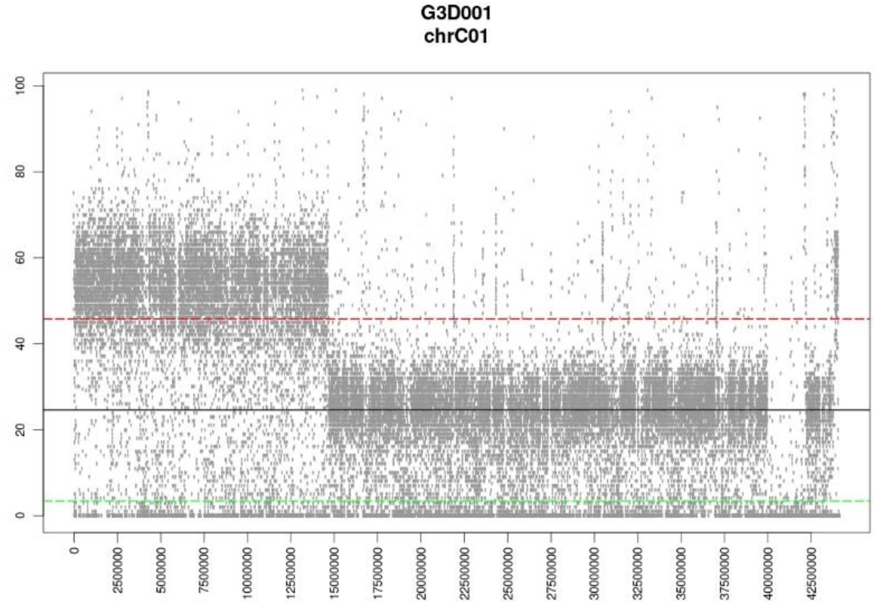

Supplementary Figure 28 Coverage of chromosome C01 from G3D001 self-pollinated ovule based on the Express 617 reference. Duplications are shown above the red line and deletions below the green line

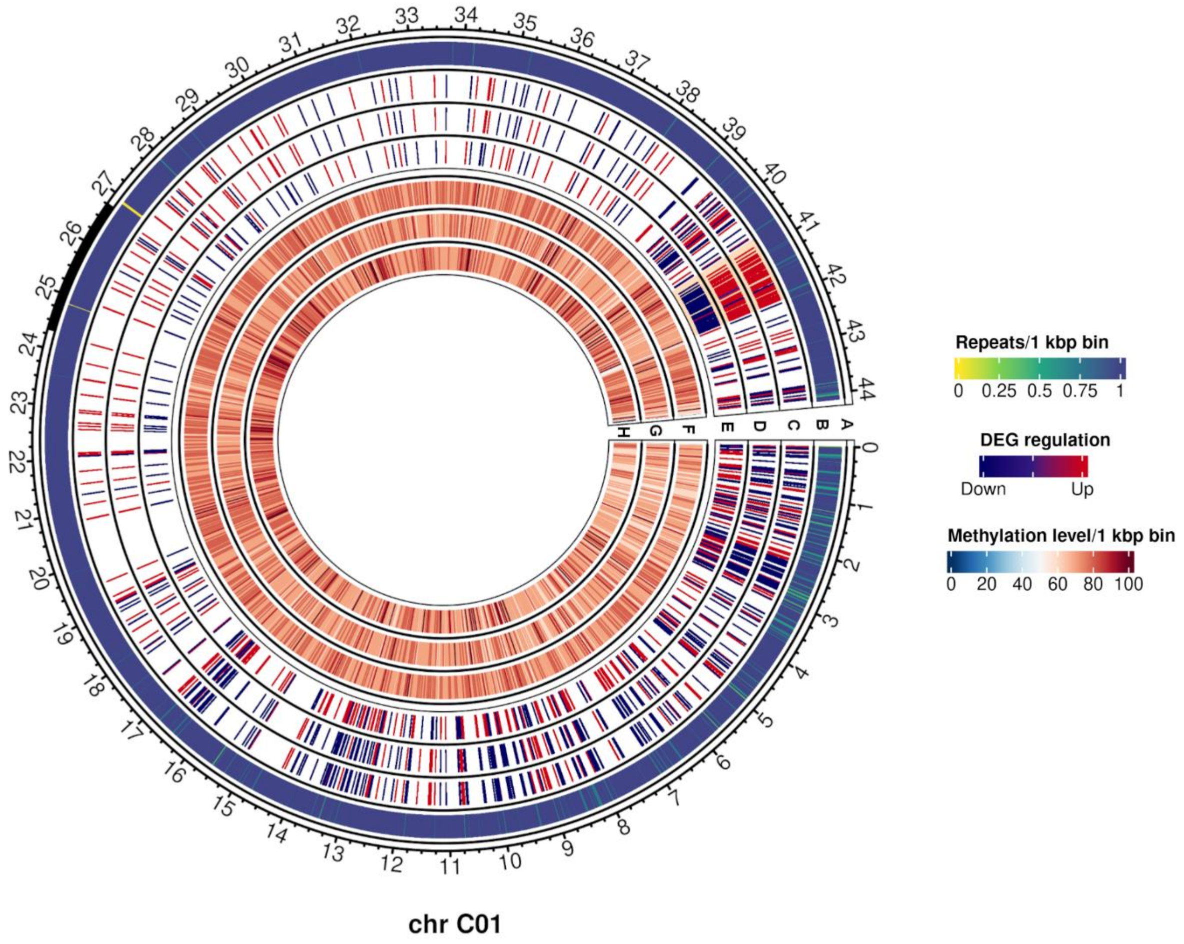

Supplementary Figure 29 Differentially expressed genes (DEGs) and methylation levels from 15 days after pollination ovules from F0 and parents in chromosome C01. Outer to inner tracks correspond to: **a** Predicted centromere positions in black; **b** Repeat density per 1 kbp bin; **c-e** DEG regulation in **(c)** Express 617, **(d)** F0 and **(e)** G3D001; **f-h:** Methylation levels per 1 kbp bin in **(f)** Express 617, **(g)** F0 and **(h)** G3D001. A differentially expressed chromosome segment between around 40.75 Mbp and 42 Mbp is highlighted in orange in tracks c-e.

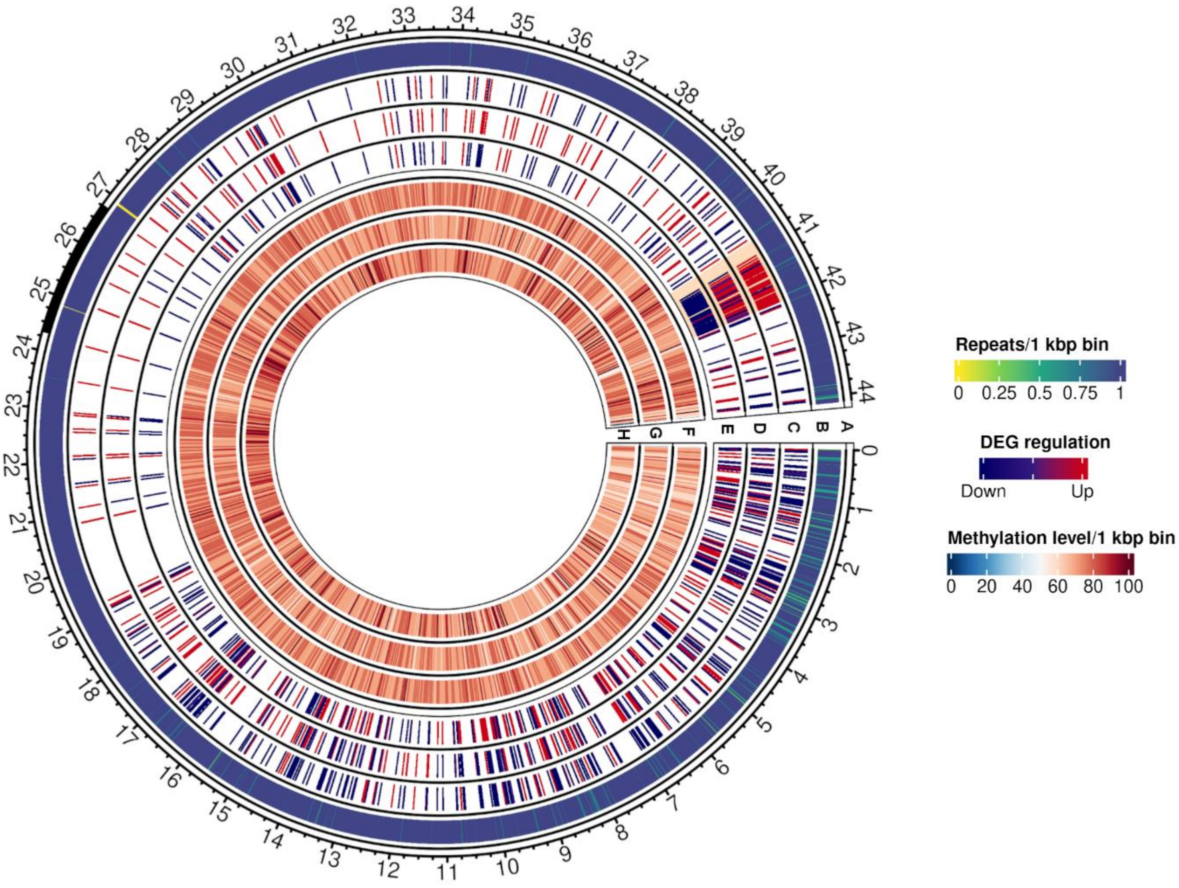

chr C01

Supplementary Figure 30 Differentially expressed genes (DEGs) and methylation levels from 15 days after pollination ovules from F1 and parents in chromosome C01. Outer to inner tracks correspond to: **a** Predicted centromere positions in black; **b** Repeat density per 1 kbp bin; **c-e** DEG regulation in **(c)** Express 617, **(d)** F1 and **(e)** G3D001; **f-h:** Methylation levels per 1 kbp bin in **(f)** Express 617, **(g)** F1 and **(h)** G3D001. A differentially expressed chromosome segment between around 40.75 Mbp and 42 Mbp is highlighted in orange in tracks c-e.

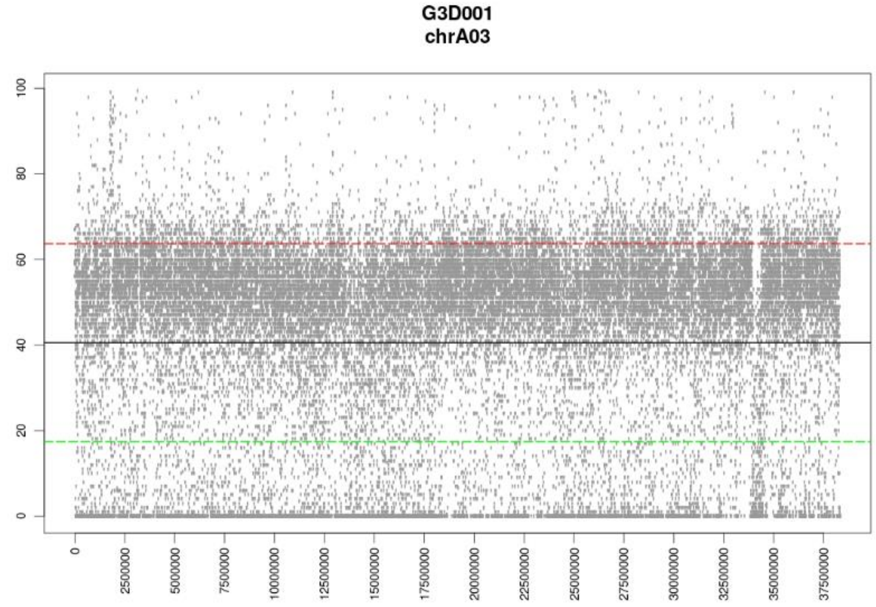

Supplementary Figure 31 Coverage of chromosome A03 from G3D001 self-pollinated ovule based on the Express 617 reference. Duplications are shown above the red line and deletions below the green line

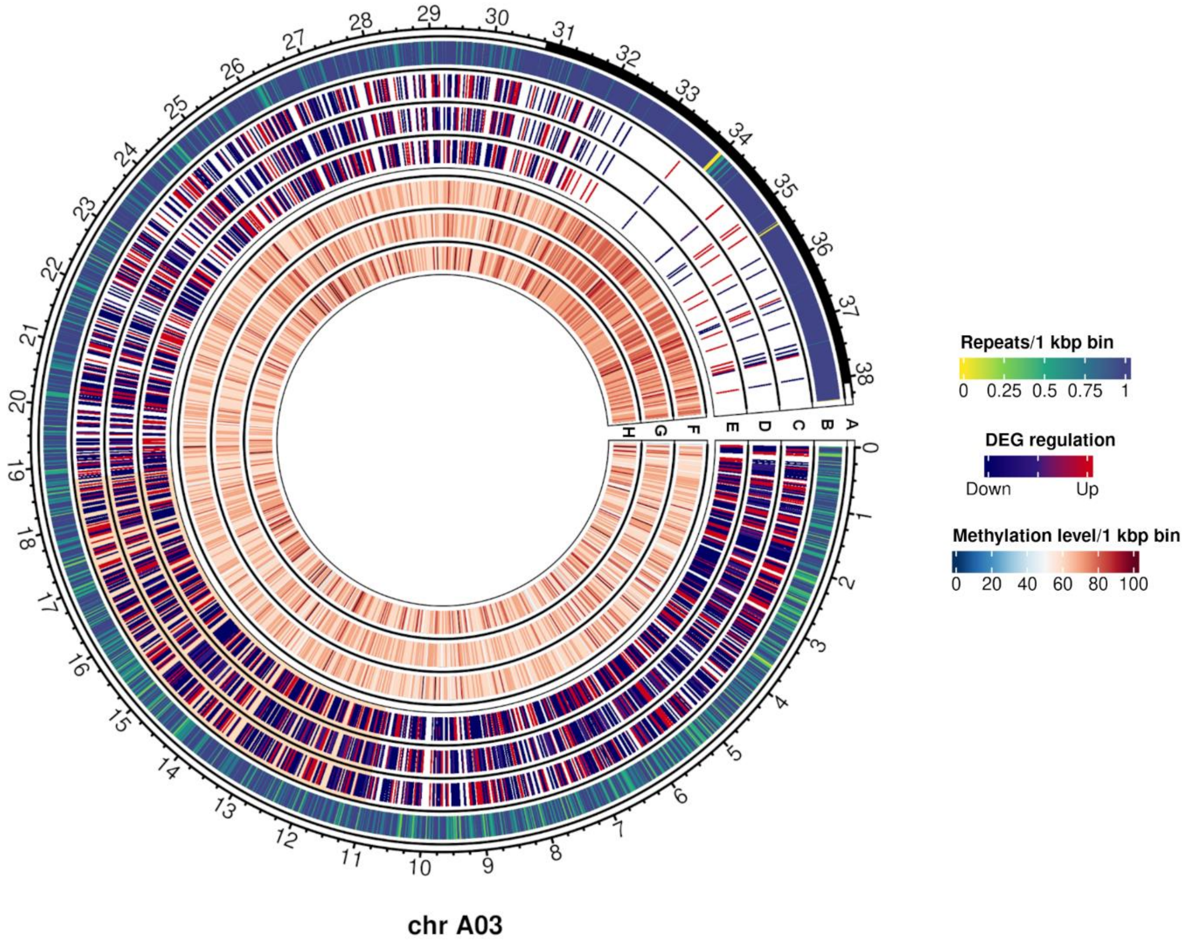

Supplementary Figure 32 Differentially expressed genes (DEGs) and methylation levels from 30 days after pollination ovules from F0 and parents in chromosome A03. Outer to inner tracks correspond to: **a** Predicted centromere positions in black; **b** Repeat density per 1 kbp bin; **c-e**, DEG regulation in **(c)** Express 617, **(d)** F0 and **(e)** G3D001; **f-h:** Methylation levels per 1 kbp bin in **(f)** Express 617, **(g)** F0 and **(h)** G3D001. A differentially expressed chromosome segment between around 11 Mbp and 18.8 Mbp is highlighted in orange in tracks c-e.

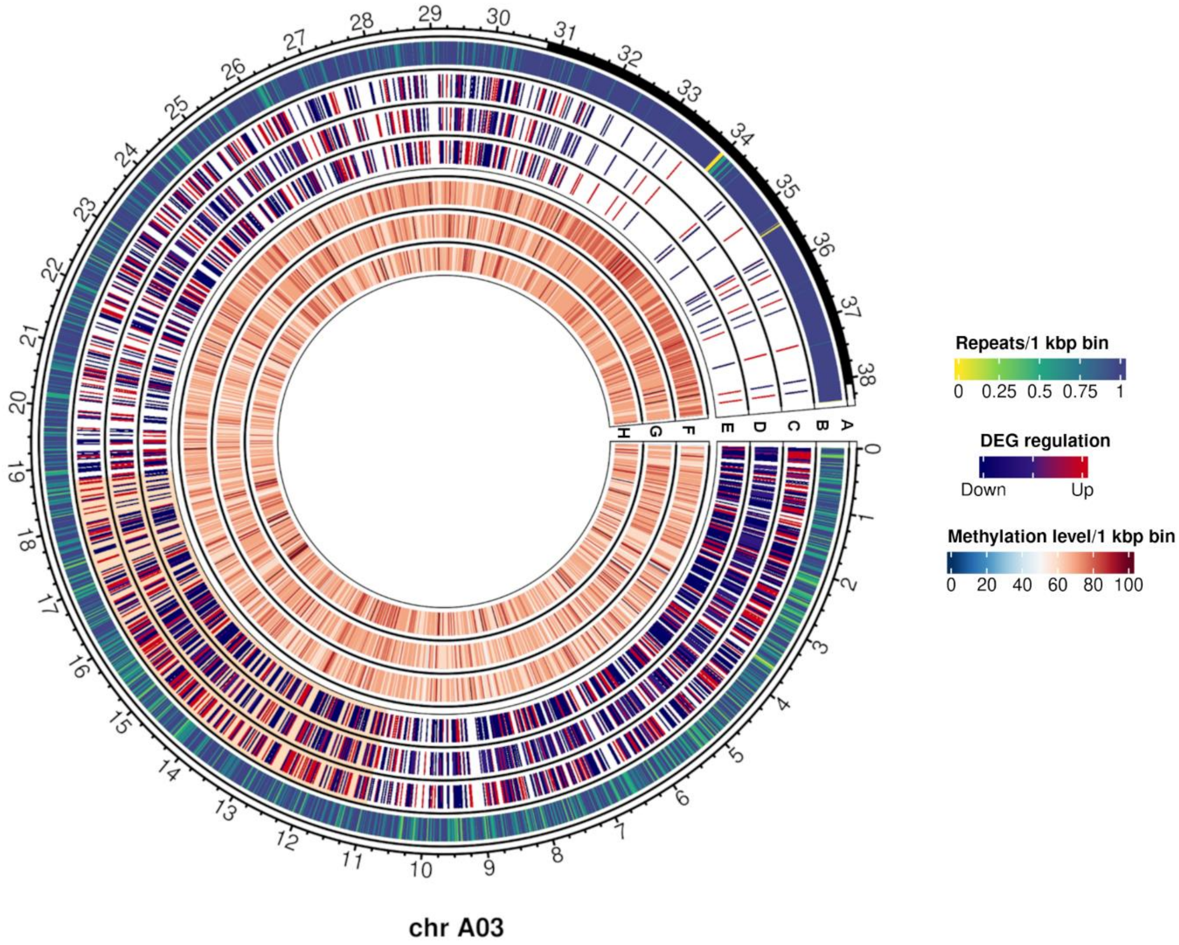

Supplementary Figure 33 Differentially expressed genes (DEGs) and methylation levels from 15 days after pollination ovules from F1 and parents in chromosome A03. Outer to inner tracks correspond to: **a** Predicted centromere positions in black; **b** Repeat density per 1 kbp bin; **c-e**, DEG regulation in **(c)** Express 617, **(d)** F1 and **(e)** G3D001; **f-h:** Methylation levels per 1 kbp bin in **(f)** Express 617, **(g)** F1 and **(h)** G3D001. A differentially expressed chromosome segment between around 11 Mbp and 18.8 Mbp is highlighted in orange in tracks c-e.

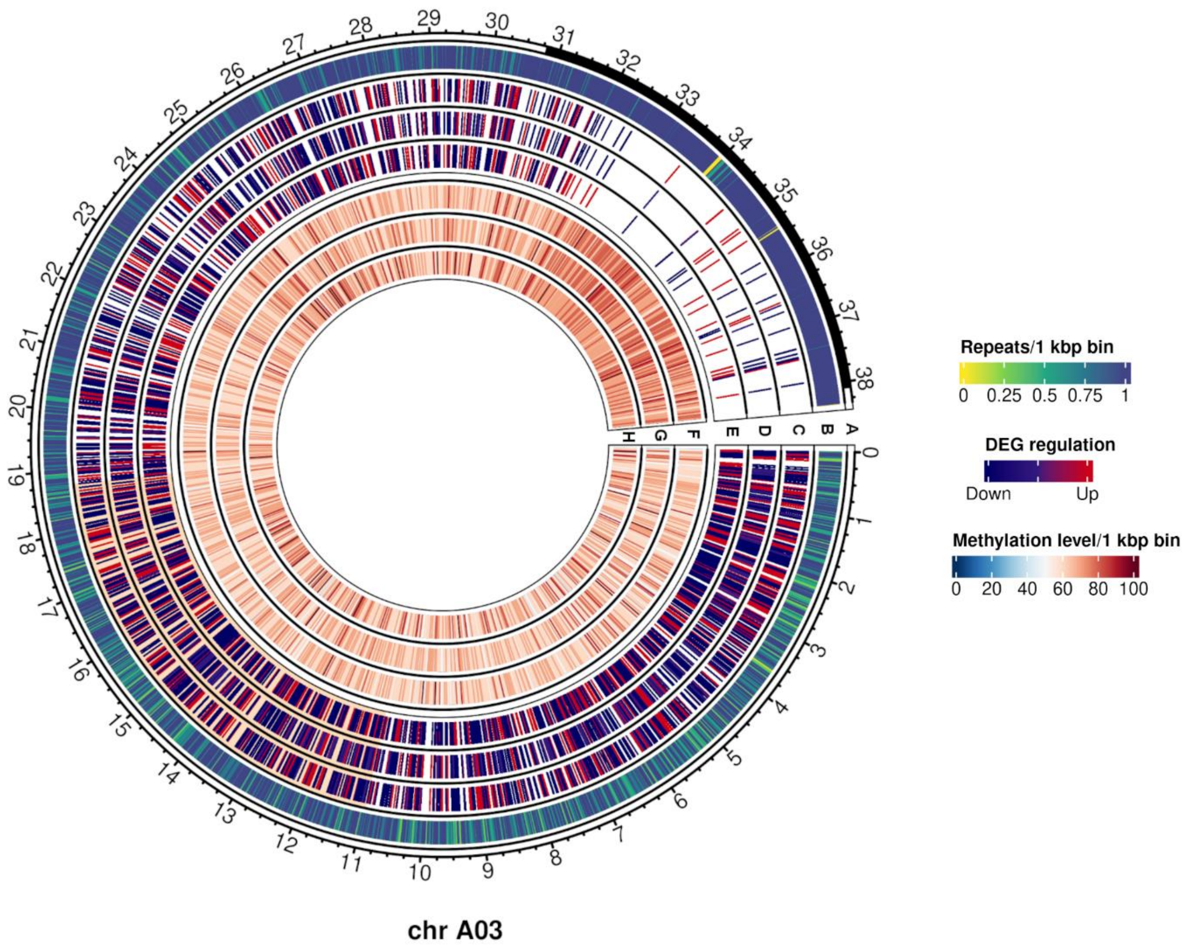

Supplementary Figure 34 Differentially expressed genes (DEGs) and methylation levels from 30 days after pollination ovules from F1 and parents in chromosome A03. Outer to inner tracks correspond to: **a** Predicted centromere positions in black; **b** Repeat density per 1 kbp bin; **c-e**, DEG regulation in **(c)** Express 617, **(d)** F1 and **(e)** G3D001; **f-h:** Methylation levels per 1 kbp bin in **(f)** Express 617, **(g)** F1 and **(h)** G3D001. A differentially expressed chromosome segment between around 11 Mbp and 18.8 Mbp is highlighted in orange in tracks c-e.
